# Amplitude Constrained Vector Gaussian Wiretap Channel: Properties of the Secrecy-Capacity-Achieving Input Distribution

**DOI:** 10.3390/e25050741

**Published:** 2023-04-30

**Authors:** Antonino Favano, Luca Barletta, Alex Dytso

**Affiliations:** 1 Dipartimento di Elettronica, Informazione e Bioingegneria, Politecnico di Milano, 20133 Milano, Italy; 2 Qualcomm, Bridgewater, NJ 08807, USA

**Keywords:** wiretap channel, MIMO, amplitude constraints

## Abstract

This paper studies the secrecy capacity of an *n*-dimensional Gaussian wiretap channel under a peak power constraint. This work determines the largest peak power constraint ${\bar{R}}_{n}$, such that an input distribution uniformly distributed on a single sphere is optimal; this regime is termed the low-amplitude regime. The asymptotic value of ${\bar{R}}_{n}$ as *n* goes to infinity is completely characterized as a function of noise variance at both receivers. Moreover, the secrecy capacity is also characterized in a form amenable to computation. Several numerical examples are provided, such as the example of the secrecy-capacity-achieving distribution beyond the low-amplitude regime. Furthermore, for the scalar case (n=1), we show that the secrecy-capacity-achieving input distribution is discrete with finitely many points at most at the order of $\frac{R^{2}}{\sigma_{1}^{2}}$, where $\sigma_{1}^{2}$ is the variance of the Gaussian noise over the legitimate channel.

## 1. Introduction

Consider the vector Gaussian wiretap channel with outputs

(1a) $$\begin{array}{cc} Y_{1} & =X+N_{1}, \end{array}$$

(1b) $$\begin{array}{cc} Y_{2} & =X+N_{2}, \end{array}$$

where $X\in R^{n}$, $N_{1}∼N\left(0_{n},\sigma_{1}^{2}I_{n}\right)$ and $N_{2}∼N\left(0_{n},\sigma_{2}^{2}I_{n}\right)$, and with $\left(X,N_{1},N_{2}\right)$ being mutually independent. The output $Y_{1}$ is observed by the legitimate receiver, whereas the output $Y_{2}$ is observed by the malicious receiver. In this work, we are interested in the scenario where the input X is limited by a peak power constraint or amplitude constraint, and assume that $X\in B_{0}\left(R\right)=\left\{x:\;∥x∥\leq R\right\}$, i.e., $B_{0}\left(R\right)$ is an *n*-ball centered at the origin and of radius R. For this setting, the secrecy capacity is given by

(2) $$\begin{array}{cc} C_{s}\left(\sigma_{1}^{2},\sigma_{2}^{2},R,n\right) & =\max_{X\in B_{0}\left(R\right)}I\left(X;Y_{1}\right)-I\left(X;Y_{2}\right) \end{array}$$

(3) $$\begin{array}{cc} \; & =\max_{X\in B_{0}\left(R\right)}I\left(X;Y_{1}|Y_{2}\right), \end{array}$$

where the last expression holds due to the (stochastically) degraded nature of the channel. It can be shown that for $\sigma_{1}^{2}\geq \sigma_{2}^{2}$ the secrecy capacity is equal to zero. Therefore, in the remainder, we assume that $\sigma_{1}^{2}<\sigma_{2}^{2}$.

We are interested in studying the input distribution $P_{X^{★}}$ that maximizes (3) in the low (but not vanishing) amplitude regime. Since closed-form expressions for secrecy capacity are rare, we derive the secrecy capacity in an integral form that is easy to evaluate. For the scalar case (n=1), we establish an upper bound on the number of mass points of $P_{X^{★}}$, valid for any amplitude regime. We also argue in Section 2.3 that the solution to the secrecy capacity can shed light on other problems seemingly unrelated to security. The paper also provides a number of numerical simulations of $P_{X^{★}}$ and $C_{s}$, the data for which are made available at [1].

### 1.1. Literature Review

The wiretap channel was introduced by Wyner in [2], who also established the secrecy capacity of the degraded wiretap channel. The results of [2] were extended to the Gaussian wiretap channel in [3]. The wiretap channel plays a central role in network information theory; the interested reader is referred to [4,5,6,7,8] and references therein for a detailed treatment of the topic. Furthermore, for an in-depth discussion on the wiretap fading channel, refer to [9,10,11,12].

In [3], it was shown that the secrecy-capacity-achieving input distribution of the Gaussian wiretap channel, under an average power constraint, is Gaussian. In [13], the authors investigated the Gaussian wiretap channel consisting of two antennas, both at the transmitter and receiver sides, and of a single antenna for the eavesdropper. The secrecy capacity of the MIMO wiretap channel was characterized in [14,15], where the Gaussian input was shown to be optimal. An elegant proof, using the I-MMSE relationship [16], of the optimality of Gaussian input, is given in [17]. Moreover, an alternative approach in the characterization of the secrecy capacity of a MIMO wiretap channel was proposed in [18]. In [19,20], the authors discuss the optimal signaling for secrecy rate maximization under average power constraints.

The secrecy capacity of the Gaussian wiretap channel under the peak power constraint has received far less attention. The secrecy capacity of the scalar Gaussian wiretap channel with an amplitude and power constraint was considered in [21], where the authors showed that the capacity-achieving input distribution $P_{X^{★}}$ is discrete with finitely many support points.

The work of [21] was extended to noise-dependent channels by Soltani and Rezki in [22]. For further studies on the properties of the secrecy-capacity-achieving input distribution for a class of degraded wiretap channels, refer to [23,24,25].

The secrecy capacity for the vector wiretap channel with a peak power constraint was considered in [25], where it was shown that the optimal input distribution is concentrated on finitely many co-centric shells.

### 1.2. Contributions and Paper Outline

In Section 2, we introduce the mathematical tools, assumptions, and definitions used throughout the paper. Specifically, in Section 2.1, we introduce the oscillation theorem. In Section 2.2, we give a definition of low-amplitude regimes. Moreover, in Section 2.3, we show how the wiretap channel can be seen as a generalization of point-to-point channels and the evaluation of the largest minimum mean square error (MMSE), both under the assumption of amplitude-constrained input. In Section 2.4, we provide a definition of the Karush–Kuhn–Tucker (KKT) conditions for the wiretap channel.

In Section 3, we detail our main results. Theorem 2 provides a sufficient condition for the optimality of a single hypersphere. Theorem 3 and Theorem 4 give the conditions under which we can fully characterize the behavior of ${\bar{R}}_{n}$, that is, the radius below which we are in the low-amplitude regime, i.e., the optimal input distribution is composed of a single shell. Furthermore, Theorem 5 gives an implicit and an explicit upper bound on the number of mass points of the secrecy-capacity-achieving input distribution when n=1.

In Section 4, we derive the secrecy capacity expression for the low-amplitude regime in Theorem 6. We also investigate its behavior when the number of antennas *n* goes to infinity.

Section 5 extends the investigation of the secrecy capacity beyond the low-amplitude regime. We numerically estimate both the optimal input pmf and the resulting capacity via an algorithmic procedure based on the KKT conditions introduced in Lemma 2.

Section 6, Section 7, Section 8 and Section 9 provide the proof for Theorem 3 and Theorem 4–6, respectively. Finally, Section 10 concludes the paper.

### 1.3. Notation

We use bold letters for vectors (x) and uppercase letters for random variables (*X*). We denote by ∥x∥ the Euclidean norm of the vector x. Given a vector $x\in R^{n}$ and a scalar *a*, with a little abuse of notation, we denote $∥a\cdot e_{1}+x∥$ by ∥a+x∥, where $e_{1}=\left[1,0,\cdots ,0\right]$ is the first vector in the standard basis of the Euclidean vector space $R^{n}$. Given a random variable *X*, its probability density function (pdf), pmf, and cumulative distribution function are denoted by $f_{X}$, $P_{X}$, and $F_{X}$, respectively. The support set of $P_{X}$ is denoted and defined as

(4) $$\begin{array}{cc} \mathrm{supp}(P_{X}) & =\{x:\;\mathrm{for}\;\mathrm{every}\;\mathrm{open}\;\mathrm{set}\;D∋x\;\mathrm{we}\;\mathrm{have}\;\mathrm{that}\;P_{X}\left(D\right)>0\}. \end{array}$$

We denote by N(μ,Σ) a multivariate Gaussian distribution with mean vector μ and covariance matrix Σ. The pdf of a Gaussian random variable with zero mean and variance $\sigma^{2}$ is denoted by $\phi_{\sigma }\left(\cdot \right)$. We denote by $\chi_{n}^{2}\left(\lambda \right)$ the noncentral chi-square distribution with *n* degrees of freedom and with noncentrality parameter λ. We represent the n×1 vector of zeros by $0_{n}$ and the n×n identity matrix by $I_{n}$. Furthermore, we represent by D the relative entropy. The minimum mean squared error is denoted by

(5) $$\begin{array}{c} \mathrm{mmse}\left(X|X+N\right)=E\left[{∥X-E\left[X|X+N\right]∥}^{2}\right]. \end{array}$$

The modified Bessel function of the first kind of order v≥0 is denoted by $I_{v}\left(x\right),x\in R$. The following ratio of the Bessel functions is commonly used in this work:

(6) $$h_{v}\left(x\right)=\frac{I_{v}\left(x\right)}{I_{v-1}\left(x\right)},\;x\in R,\;v\geq 0.$$

Finally, the number of zeros (counted in accordance with their multiplicities) of a function f:R→R on the interval I is denoted by N(I,f). Similarly, if f:C→C is a function on the complex domain, N(D,f) denotes the number of its zeros within the region D.

## 2. Preliminaries

### 2.1. Oscillation Theorem

In this work, we often need to upper bound the number of oscillations of a function, i.e., its number of sign changes. This is useful, for example, to bound the number of zeros of a function or the number of roots of an equation. To be more precise, let us define the number of sign changes as follows.

**Definition** **1** (Sign Changes of a Function). *The number of sign changes of a function ξ:Ω→R is given by*

(7) $$S\left(\xi \right)=\underset{m\in N}{\sup}\left\{\underset{y_{1}<\cdots <y_{m}\subseteq \Omega }{\sup}N{\left\{\xi \left(y_{i}\right)\right\}}_{i=1}^{m}\right\},$$

*where $N{\left\{\xi \left(y_{i}\right)\right\}}_{i=1}^{m}$ is the number of sign changes of the sequence ${\left\{\xi \left(y_{i}\right)\right\}}_{i=1}^{m}$.*

**Definition** **2** (Totally Positive Kernel). *A function $f:I_{1}\times I_{2}\to R$ is said to be a totally positive kernel of order n if $\det\left({\left[f\left(x_{i},y_{j}\right)\right]}_{i,j=1}^{m}\right)>0$ for all 1≤m≤n, for all $x_{1}<\cdots <x_{m}\in I_{1}$, and $y_{1}<\cdots <y_{m}\in I_{2}$. If f is a totally positive kernel of order n for all n∈N, then f is a strictly totally positive kernel.*

In [26], Karlin noticed that some integral transformations have a *variation-diminishing* property, which is described in the following theorem.

**Theorem** **1** (Oscillation Theorem). *Given domains $I_{1}$ and $I_{2}$, let $p:I_{1}\times I_{2}\to R$ be a strictly totally positive kernel. For an arbitrary y, suppose $p\left(\cdot ,y\right):I_{1}\to R$ is an n-times differentiable function. Assume that μ is a measure on $I_{2}$, and let $\xi :I_{2}\to R$ be a function with S(ξ)=n. For $x\in I_{1}$, define*

(8) Ξ(x)=∫ξ(y)p(x,y)dμ(y).

*If $\Xi :I_{1}\to R$ is an n-times differentiable function, then either $N(I_{1},\Xi )\leq n$, or Ξ≡0.*

The above theorem says that the number of zeros of a function Ξ, which is the output of the integral transformation, is less than the number of sign changes of the function ξ, which is the input to the integral transformation.

### 2.2. Low-Amplitude Regime

In this work, a low-amplitude regime is defined as follows.

**Definition** **3.** *Let $X_{R}∼P_{X_{R}}$ be uniform on C(R)={x:∥x∥=R}. The capacity in (3) is said to be in the low-amplitude regime if $R\leq {\bar{R}}_{n}\left(\sigma_{1}^{2},\sigma_{2}^{2}\right)$, where*

(9) $${\bar{R}}_{n}\left(\sigma_{1}^{2},\sigma_{2}^{2}\right)=\max\left\{R:P_{X_{R}}=\arg\max_{P_{X}:\;X\in B_{0}\left(R\right)}I\left(X;Y_{1}|Y_{2}\right)\right\}.$$

*If the set in (9) is empty, then we assign ${\bar{R}}_{n}\left(\sigma_{1}^{2},\sigma_{2}^{2}\right)=0$.*

The quantity ${\bar{R}}_{n}\left(\sigma_{1}^{2},\sigma_{2}^{2}\right)$ represents the largest radius R, for which $P_{X_{R}}$ is secrecy-capacity-achieving.

One of the main objectives of this work is to characterize ${\bar{R}}_{n}\left(\sigma_{1}^{2},\sigma_{2}^{2}\right)$.

### 2.3. Connections to Other Optimization Problems

The distribution $P_{X_{R}}$ occurs in a variety of statistical and information-theoretic applications. For example, consider the following two optimization problems:

(10) $$\begin{array}{cc} \max_{P_{X}:\;X\in B_{0}\left(R\right)} & I(X;X+N), \end{array}$$

(11) $$\begin{array}{cc} \max_{P_{X}:\;X\in B_{0}\left(R\right)} & \mathrm{mmse}(X|X+N), \end{array}$$

where $N∼N(0_{n},\sigma^{2}I_{n})$. The first problem seeks to characterize the capacity of the point-to-point channel under an amplitude constraint, and the second problem seeks to find the largest minimum mean squared error under the assumption that the signal has bounded amplitude; the interested reader is referred to [27,28,29] for a detailed background on both problems.

Similarly to the wiretap channel, we can define the low-amplitude regime for both problems as the largest R such that $P_{X_{R}}$ is optimal and denote these by ${\bar{R}}_{n}^{\mathrm{ptp}}\left(\sigma^{2}\right)$ and ${\bar{R}}_{n}^{\mathrm{MMSE}}\left(\sigma^{2}\right)$. We now argue that both ${\bar{R}}_{n}^{\mathrm{ptp}}\left(\sigma^{2}\right)$ and ${\bar{R}}_{n}^{\mathrm{MMSE}}\left(\sigma^{2}\right)$ can be seen as a special case of the wiretap solution. Hence, the wiretap channel provides an interesting unification and generalization of these two problems.

First, note that the point-to-point solution can be recovered from the wiretap by simply specializing the wiretap channel to the point-to-point channel, that is,

(12) $$\begin{array}{c} {\bar{R}}_{n}^{\mathrm{ptp}}\left(\sigma^{2}\right)=\lim_{\sigma_{2}\to \infty }{\bar{R}}_{n}\left(\sigma^{2},\sigma_{2}^{2}\right). \end{array}$$

Second, to see that the MMSE solution can be recovered from the wiretap, recall that by the I-MMSE relationship [16] we have that

$$\begin{array}{cc} \; & \max_{P_{X}:\;X\in B_{0}\left(R\right)}I\left(X;Y_{1}\right)-I\left(X;Y_{2}\right) \end{array}$$

(13) $$\begin{array}{cc} \; & =\max_{P_{X}:\;X\in B_{0}\left(R\right)}\frac{1}{2}\int_{\sigma_{1}^{2}}^{\infty }\frac{\mathrm{mmse}(X|X+\sqrt{s}Z)}{s^{2}}ds-\frac{1}{2}\int_{\sigma_{2}^{2}}^{\infty }\frac{\mathrm{mmse}(X|X+\sqrt{s}Z)}{s^{2}}ds \end{array}$$

(14) $$\begin{array}{cc} \; & =\max_{P_{X}:\;X\in B_{0}\left(R\right)}\frac{1}{2}\int_{\sigma_{1}^{2}}^{\sigma_{2}^{2}}\frac{\mathrm{mmse}(X|X+\sqrt{s}Z)}{s^{2}}ds \end{array}$$

where Z is standard Gaussian. Now, note that if we choose $\sigma_{2}^{2}=\sigma_{1}^{2}+\epsilon$, then by the mean value theorem we arrive at

(15) $$\begin{array}{cc} \; & \max_{P_{X}:\;X\in B_{0}\left(R\right)}I\left(X;Y_{1}\right)-I\left(X;Y_{2}\right)=\max_{P_{X}:\;X\in B_{0}\left(R\right)}\frac{\epsilon }{2}\frac{\mathrm{mmse}(X|X+\sqrt{\sigma_{1}^{2}}Z)}{\sigma_{1}^{4}}+o\left(\epsilon \right), \end{array}$$

where $\lim_{\epsilon \to 0^{+}}o\left(\epsilon \right)/\epsilon =0$. Consequently, for a small enough ϵ>0,

(16) $${\bar{R}}_{n}^{\mathrm{MMSE}}\left(\sigma^{2}\right)={\bar{R}}_{n}\left(\sigma^{2},\sigma^{2}+\epsilon \right).$$

### 2.4. KKT Conditions

Let us define the secrecy density for the vector Gaussian wiretap channel as

(17) $$\begin{array}{cc} \Xi (x;P_{X^{★}}) & =D\left(f_{Y_{1}|X}\left(\cdot |x\right)∥f_{Y_{1}^{★}}\right)-D\left(f_{Y_{2}|X}\left(\cdot |x\right)∥f_{Y_{2}^{★}}\right), \end{array}$$

where D(·∥·) is the relative entropy.

For the scalar case (n=1), the KKT conditions are necessary and sufficient to ensure that $P_{X^{★}}$ is capacity-achieving [21].

**Lemma** **1.** *$P_{X^{★}}$ maximizes (3) if, and only if,*

(18) $$\begin{array}{cc} \Xi (x) & =C_{s}\left(\sigma_{1}^{2},\sigma_{2}^{2},R,1\right),\;x\in \mathrm{supp}\left(P_{X^{★}}\right), \end{array}$$

(19) $$\begin{array}{cc} \Xi (x) & \leq C_{s}\left(\sigma_{1}^{2},\sigma_{2}^{2},R,1\right),\;x\in \left[-R,R\right], \end{array}$$

*where for x∈R*

(20) $$\begin{array}{cc} \Xi (x) & =D\left(f_{Y_{1}|X}\left(\cdot |x\right)∥f_{Y_{1}^{★}}\right)-D\left(f_{Y_{2}|X}\left(\cdot |x\right)∥f_{Y_{2}^{★}}\right) \end{array}$$

(21) $$\begin{array}{cc} \; & =E\left[g(Y_{1})|X=x\right]+\log\left(\frac{\sigma_{2}}{\sigma_{1}}\right), \end{array}$$

*and where*

(22) $$\begin{array}{c} g\left(y\right)=E\left[\log\frac{f_{Y_{2}^{★}}\left(y+N\right)}{f_{Y_{1}^{★}}\left(y\right)}\right],\;y\in R, \end{array}$$

*with*$N∼N(0,\sigma_{2}^{2}-\sigma_{1}^{2})$.

**Proof.**  The first part of Lemma 1 was shown in [21]. The proof of (21) goes as follows:

(23) $$\begin{array}{cc} \; & D\left(f_{Y_{1}|X}\left(\cdot |x\right)∥f_{Y_{1}^{★}}\right)-D\left(f_{Y_{2}|X}\left(\cdot |x\right)∥f_{Y_{2}^{★}}\right)-\log\left(\frac{\sigma_{2}}{\sigma_{1}}\right) \end{array}$$

(24) $$\begin{array}{cc} \; & =\int_{-\infty }^{\infty }\log\frac{1}{f_{Y_{1}^{★}}\left(y\right)}\phi_{\sigma_{1}}\left(y-x\right)dy-\int_{-\infty }^{\infty }\log\frac{1}{f_{Y_{2}^{★}}\left(y\right)}E\left[\phi_{\sigma_{1}}\left(y-x-N\right)\right]dy \end{array}$$

(25) $$\begin{array}{cc} \; & =\int_{-\infty }^{\infty }\log\frac{1}{f_{Y_{1}^{★}}\left(y\right)}\phi_{\sigma_{1}}\left(y-x\right)dy-\int_{-\infty }^{\infty }E\left[\log\frac{1}{f_{Y_{2}^{★}}\left(y+N\right)}\right]\phi_{\sigma_{1}}\left(y-x\right)dy \end{array}$$

(26) $$\begin{array}{cc} \; & =\int_{-\infty }^{\infty }E\left[\log\frac{f_{Y_{2}^{★}}\left(y+N\right)}{f_{Y_{1}^{★}}\left(y\right)}\right]\phi_{\sigma_{1}}\left(y-x\right)dy \end{array}$$

(27) $$\begin{array}{cc} \; & =\int_{-\infty }^{\infty }g\left(y\right)\phi_{\sigma_{1}}\left(y-x\right)dy, \end{array}$$

where $N∼N(0,\sigma_{2}^{2}-\sigma_{1}^{2})$ and (24) hold by noticing that $\phi_{\sigma_{2}}\left(y-x\right)$ can be reformulated as the convolution of Gaussian pdfs $E[\phi_{\sigma_{1}}\left(y-x-N\right)]$; in (25) we applied the change in variable y↦y+N. This concludes the proof.    □

The convexity of the optimization problem is also guaranteed for the vector wiretap model in (1) with n>1. Then, the results of Lemma 1 can be extended to the vector case as follows.

**Lemma** **2.** *$P_{X^{★}}$ maximizes (3) if, and only if,*

(28a) $$\begin{array}{cc} \Xi (x;P_{X^{★}}) & =C_{s}\left(\sigma_{1}^{2},\sigma_{2}^{2},R,n\right),\;x\in \mathrm{supp}\left(P_{X^{★}}\right), \end{array}$$

(28b) $$\begin{array}{cc} \Xi (x;P_{X^{★}}) & \leq C_{s}\left(\sigma_{1}^{2},\sigma_{2}^{2},R,n\right),\;x\in B_{0}\left(R\right), \end{array}$$

*where for $x\in R^{n}$*

(29) $$\begin{array}{cc} \Xi (x;P_{X^{★}}) & =D\left(f_{Y_{1}|X}\left(\cdot |x\right)∥f_{Y_{1}^{★}}\right)-D\left(f_{Y_{2}|X}\left(\cdot |x\right)∥f_{Y_{2}^{★}}\right) \end{array}$$

(30) $$\begin{array}{cc} \; & =E\left[g(Y_{1})|X=x\right], \end{array}$$

*and where*

(31) $$\begin{array}{c} g\left(y\right)=E\left[\log\frac{f_{Y_{2}^{★}}\left(y+N\right)}{f_{Y_{1}^{★}}\left(y\right)}\right]+n\log\left(\frac{\sigma_{2}}{\sigma_{1}}\right),\;y\in R^{n}, \end{array}$$

*with $N∼N(0_{n},\left(\sigma_{2}^{2}-\sigma_{1}^{2}\right)I_{n})$.*

**Proof.**  This is a straightforward vector extension of Lemma 1.    □

Thanks to the spherical symmetry of the additive noise distributions and of $P_{X}$, the secrecy density $\Xi (x;P_{X})$ can be expressed as a function of ∥x∥ only. Therefore, we denote the secrecy density in spherical coordinates by $\tilde{\Xi }(∥x∥;P_{∥X∥})$, and give a rigorous definition in (A9).

## 3. Main Results

### 3.1. A New Sufficient Condition on the Optimality of $P_{X_{R}}$

Our first main result provides a sufficient condition for the optimality of $P_{X_{R}}$.

**Theorem** **2.** *If*

(32) $$R<\sigma_{1}^{2}\sqrt{n\left(\frac{1}{\sigma_{1}^{2}}-\frac{1}{\sigma_{2}^{2}}\right)},$$

*then $P_{X_{R}}$ is secrecy-capacity-achieving.*

**Proof.**  Let us consider the equivalent definition of the secrecy density in spherical coordinates (A9). Note that if the derivative of $\tilde{\Xi }(∥x∥;P_{∥X_{R}∥})$ makes at most one sign change, from negative to positive, then the maximum of $∥x∥\mapsto \tilde{\Xi }(∥x∥;P_{∥X_{R}∥})$ occurs at either ∥x∥=0 or ∥x∥=R.

From Lemma A1 in the Appendix B, the derivative of $\tilde{\Xi }$ is as given below

(33) $$\begin{array}{c} {\tilde{\Xi }}^{\prime }(∥x∥;P_{∥X_{R}∥})=∥x∥\;E\left[{\tilde{M}}_{2}\left(\sigma_{1}Q_{n+2}\right)-M_{1}\left(\sigma_{1}Q_{n+2}\right)\right] \end{array}$$

where $Q_{n+2}^{2}$ is a noncentral chi-square random variable with n+2 degrees of freedom and noncentrality parameter $\frac{{∥x∥}^{2}}{\sigma_{1}^{2}}$, and

(34) $$\begin{array}{cc} M_{i}\left(y\right) & =\frac{1}{\sigma_{i}^{2}}\left(\frac{R}{y}h_{\frac{n}{2}}\left(\frac{R}{\sigma_{i}^{2}}y\right)-1\right),\;i\in \left\{1,2\right\} \end{array}$$

(35) $$\begin{array}{cc} {\tilde{M}}_{2}\left(y\right) & =E\left[M_{2}(∥y+W∥)\right], \end{array}$$

where $W∼N(0_{n+2},\left(\sigma_{2}^{2}-\sigma_{1}^{2}\right)I_{n+2})$. A calculation related to (33) was erroneously performed in [27]. However, this error does not change the results of [27] as only the sign of the derivative is important and not the value itself. Note that ${\tilde{\Xi }}^{\prime }\left(0;P_{∥X_{R}∥}\right)=0$ and that ${\tilde{\Xi }}^{\prime }(∥x∥;P_{∥X_{R}∥})>0$ for a sufficiently large ∥x∥; in fact, we have

(36) $$\begin{array}{cc} {\tilde{\Xi }}^{\prime }(∥x∥;P_{∥X_{R}∥}) & >∥x∥\left(\frac{1}{\sigma_{1}^{2}}-\frac{1}{\sigma_{2}^{2}}\right)-\frac{∥x∥}{\sigma_{1}^{2}}E\left[\frac{R}{\sigma_{1}Q_{n+2}}\right] \end{array}$$

(37) $$\begin{array}{cc} \; & =∥x∥\left(\frac{1}{\sigma_{1}^{2}}-\frac{1}{\sigma_{2}^{2}}\right)-\frac{∥x∥}{\sigma_{1}^{2}}E\left[\frac{R}{∥x∥}h_{\frac{n}{2}}\left(\frac{∥x∥}{\sigma_{1}}Q_{n}\right)\right] \end{array}$$

(38) $$\begin{array}{cc} \; & \geq ∥x∥\left(\frac{1}{\sigma_{1}^{2}}-\frac{1}{\sigma_{2}^{2}}\right)-\frac{R}{\sigma_{1}^{2}}, \end{array}$$

where (36) follows from $0\leq h_{\frac{n}{2}}\left(x\right)\leq 1$ for x≥0; (37) follows by noticing that $\frac{R}{\sigma_{1}\sqrt{t}}f_{Q_{n+2}^{2}}\left(t\right)=\frac{R}{∥x∥}h_{\frac{n}{2}}\left(\frac{∥x∥}{\sigma_{1}}\sqrt{t}\right)f_{Q_{n}^{2}}\left(t\right)$; and finally, (38) holds by $h_{\frac{n}{2}}\left(x\right)\leq 1$.

Then, to show that $\tilde{\Xi }(∥x∥;P_{∥X_{R}∥})$ is maximized in ∥x∥=R, we need to prove that ${\tilde{\Xi }}^{\prime }(∥x∥;P_{∥X_{R}∥})$ changes sign at most once. To that end, we need Karlin’s oscillation theorem presented in Section 2.1. By using (33), the fact that the pdf of a chi-square is a positive defined kernel [26], and Theorem 1, the number of sign changes of ${\tilde{\Xi }}^{\prime }(∥x∥;P_{∥X_{R}∥})$ is upper-bounded by the number of sign changes of

(39) $$G_{\sigma_{1},\sigma_{2},R,n}\left(y\right)={\tilde{M}}_{2}\left(y\right)-M_{1}\left(y\right),$$

for $y\in R^{+}$. Note that

(40) $$\begin{array}{cc} G_{\sigma_{1},\sigma_{2},R,n}\left(y\right) & \geq -\frac{1}{\sigma_{2}^{2}}+\frac{1}{\sigma_{1}^{2}}-\frac{R}{\sigma_{1}^{2}y}h_{\frac{n}{2}}\left(\frac{R}{\sigma_{1}^{2}}y\right) \end{array}$$

(41) $$\begin{array}{cc} \; & \geq -\frac{1}{\sigma_{2}^{2}}+\frac{1}{\sigma_{1}^{2}}-\frac{R^{2}}{\sigma_{1}^{4}n}, \end{array}$$

where the inequality in (40) follows from $h_{\frac{n}{2}}\left(x\right)\geq 0$ for x≥0, and (41) follows from $h_{\frac{n}{2}}\left(x\right)\leq \frac{x}{n}$ for x≥0 and n∈N. We conclude by noting that (41) is nonnegative, hence has no sign change, for

(42) $$R<\sigma_{1}^{2}\sqrt{n\left(\frac{1}{\sigma_{1}^{2}}-\frac{1}{\sigma_{2}^{2}}\right)}$$

for all $y\in R^{+}$, thus guaranteeing that $P_{X_{R}}$ is secrecy-capacity-achieving.    □

**Remark** **1.** *As a consequence of the proof of Theorem 2, for any $R\geq 0,\sigma_{2}\geq \sigma_{1}\geq 0$ and n∈N, if $G_{\sigma_{1},\sigma_{2},R,n}\left(y\right)$ has at most one sign change, then $P_{X_{R}}$ is secrecy-capacity-achieving if, and only if, for all ∥x∥=R*

(43) $$\Xi \left(0;P_{X_{R}}\right)\leq \Xi \left(x;P_{X_{R}}\right).$$

*Because of the difficulty in evaluating analytical properties of (39), proving that $G_{\sigma_{1},\sigma_{2},R,n}$ has at most one sign change does not seem easy. However, in Appendix A, we show via extensive numerical evaluations that $G_{\sigma_{1},\sigma_{2},R,n}$ changes sign at most once for any $n,R,\sigma_{1},\sigma_{2}$ that we tried.*

### 3.2. Characterizing the Low-Amplitude Regime

Let us characterize the low-amplitude regime as follows.

**Theorem** **3.** *Consider a function*

(44) $$\begin{array}{cc} f(R)\; & =\int_{\sigma_{1}^{2}}^{\sigma_{2}^{2}}\frac{E\left[h_{\frac{n}{2}}^{2}\left(\frac{∥\sqrt{s}Z∥R}{s}\right)+h_{\frac{n}{2}}^{2}\left(\frac{∥R+\sqrt{s}Z∥R}{s}\right)\right]-1}{s^{2}}ds \end{array}$$

*where $Z∼N(0_{n},I_{n})$. If $G_{\sigma_{1},\sigma_{2},R,n}$ of (39) has at most one sign change, the input $X_{R}$ is secrecy-capacity-achieving if, and only if, $R\leq {\bar{R}}_{n}\left(\sigma_{1}^{2},\sigma_{2}^{2}\right)$, where ${\bar{R}}_{n}\left(\sigma_{1}^{2},\sigma_{2}^{2}\right)$ is given as the solution of*

(45) f(R)=0.

**Remark** **2.** *Note that (45) always has a solution. To see this, observe that $f\left(0\right)=\frac{1}{\sigma_{2}^{2}}-\frac{1}{\sigma_{1}^{2}}<0$ and $f\left(\infty \right)=\frac{1}{\sigma_{1}^{2}}-\frac{1}{\sigma_{2}^{2}}>0$. Moreover, the solution is unique because f(R) monotonically increases for R≥0.*

The solution to (45) needs to be found numerically. To avoid any loss of accuracy in the numerical evaluation of $h_{v}\left(x\right)$ for large values of *x*, we used the exponential scaling provided in the MATLAB implementation of $I_{v}\left(x\right)$. Since evaluating f(R) is rather straightforward and not time-consuming, we opted for a binary search algorithm.

In Table 1, we show the values of ${\bar{R}}_{n}\left(1,\sigma_{2}^{2}\right)$ for some values of $\sigma_{2}^{2}$ and *n*. Moreover, we report the values of ${\bar{R}}_{n}^{\mathrm{ptp}}\left(1\right)$ and ${\bar{R}}_{n}^{\mathrm{MMSE}}\left(1\right)$ from [27] in the first and the last row, respectively. As predicted by (12), we can appreciate the close match of the ${\bar{R}}_{n}^{\mathrm{ptp}}\left(1\right)$ row with the one of ${\bar{R}}_{n}\left(1,1000\right)$. Similarly, the agreement between the ${\bar{R}}_{n}^{\mathrm{MMSE}}\left(1\right)$ row and the ${\bar{R}}_{n}\left(1,1.001\right)$ row is justified by (16).

### 3.3. Large *n* Asymptotics

We now use the result in Theorem 3 to characterize the asymptotic behavior of ${\bar{R}}_{n}\left(\sigma_{1}^{2},\sigma_{2}^{2}\right)$. In particular, it is shown that ${\bar{R}}_{n}\left(\sigma_{1}^{2},\sigma_{2}^{2}\right)$ increases as $\sqrt{n}$.

**Theorem** **4.** *For $\sigma_{1}^{2}\leq \sigma_{2}^{2}$*

(46) $$\lim_{n\to \infty }\frac{{\bar{R}}_{n}\left(\sigma_{1}^{2},\sigma_{2}^{2}\right)}{\sqrt{n}}=c\left(\sigma_{1}^{2},\sigma_{2}^{2}\right),$$

*where $c=c(\sigma_{1}^{2},\sigma_{2}^{2})$ is the solution of*

(47) $$\int_{\sigma_{1}^{2}}^{\sigma_{2}^{2}}\frac{\frac{c^{2}}{{\left(\frac{\sqrt{s}}{2}+\sqrt{\frac{s}{4}+c^{2}}\right)}^{2}}+\frac{c^{2}\left(c^{2}+s\right)}{{\left(\frac{s}{2}+\sqrt{\frac{s^{2}}{4}+c^{2}\left(c^{2}+s\right)}\right)}^{2}}-1}{s^{2}}ds=0.$$

**Proof.**  See Section 7.    □

**Figure 1:**
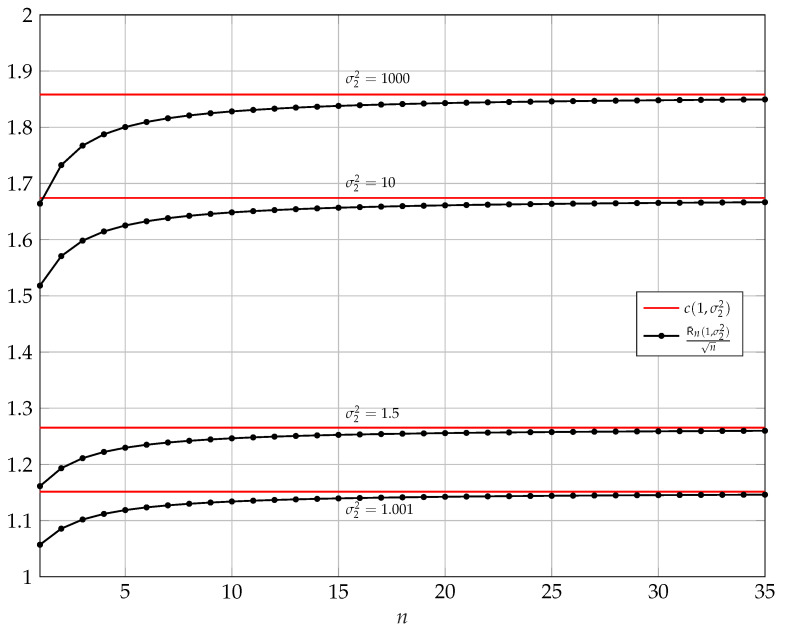
Asymptotic behavior of ${\bar{R}}_{n}\left(1,\sigma_{2}^{2}\right)/\sqrt{n}$ versus *n* for $\sigma_{1}^{2}=1$ and $\sigma_{2}^{2}=1.001,1.5,10,1000$. In red, we show $c(1,\sigma_{2}^{2})$ defined in (46).

In Figure 1, for $\sigma_{1}^{2}=1$ and $\sigma_{2}^{2}=1.001,1.5,10,1000$, we show the behavior of ${\bar{R}}_{n}\left(1,\sigma_{2}^{2}\right)/\sqrt{n}$ and how its asymptotic converges to $c(1,\sigma_{2}^{2})$.

### 3.4. Scalar Case (n=1)

For the scalar case, the optimal input distribution $P_{X^{★}}$ is discrete. In this regime, we provide an implicit and an explicit upper bound on the number of support points of the optimal input probability mass function (pmf) $P_{X^{★}}$.

**Theorem** **5.** *Let $Y_{1}^{★}$ and $Y_{2}^{★}$ be the secrecy-capacity-achieving output distributions at the legitimate and malicious receivers, respectively, and let*

(48) $$\begin{array}{c} g\left(y\right)=E\left[\log\frac{f_{Y_{2}^{★}}\left(y+N\right)}{f_{Y_{1}^{★}}\left(y\right)}\right],\;y\in R, \end{array}$$

*with $N∼N(0,\sigma_{2}^{2}-\sigma_{1}^{2})$. For R>0, an implicit upper bound on the number of support points of $P_{X^{★}}$ is*

(49) $$\begin{array}{c} |\mathrm{supp}\left(P_{X^{★}}\right)|\leq N\left(\left[-L,L\right],g\left(\cdot \right)+\kappa_{1}\right)<\infty \end{array}$$

*where*

(50) $$\begin{array}{cc} \kappa_{1} & =\log\left(\frac{\sigma_{2}}{\sigma_{1}}\right)-C_{s}, \end{array}$$

(51) $$\begin{array}{cc} L & =R\frac{\sigma_{2}+\sigma_{1}}{\sigma_{2}-\sigma_{1}}+\sqrt{\frac{\frac{\sigma_{2}^{2}-\sigma_{1}^{2}}{\sigma_{2}^{2}}+2C_{s}}{\frac{1}{\sigma_{1}^{2}}-\frac{1}{\sigma_{2}^{2}}}}. \end{array}$$

*Moreover, an explicit upper bound on the number of support points of $P_{X^{★}}$ is obtained by using*

(52) $$\begin{array}{c} N\left(\left[-L,L\right],g\left(\cdot \right)+\kappa_{1}\right)\leq \rho \frac{R^{2}}{\sigma_{1}^{2}}+O\left(\log\left(R\right)\right), \end{array}$$

*where $\rho ={\left(2e+1\right)}^{2}{\left(\frac{\sigma_{2}+\sigma_{1}}{\sigma_{2}-\sigma_{1}}\right)}^{2}+{\left(\frac{\sigma_{2}+\sigma_{1}}{\sigma_{2}-\sigma_{1}}+1\right)}^{2}$.*

The upper bounds in Theorem 5 are generalizations of the upper bounds on the number of points presented in [30] in the context of a point-to-point AWGN channel with an amplitude constraint. Indeed, if we let $\sigma_{2}\to \infty$, while keeping $\sigma_{1}$ and R fixed, then the wiretap channel reduces to the AWGN point-to-point channel.

To find a lower bound on the number of mass points, a possible approach consists of the following steps:

(53) $$\begin{array}{cc} C_{s}\left(\sigma_{1}^{2},\sigma_{2}^{2},R,1\right) & =I\left(X^{★};Y_{1}\right)-I\left(X^{★};Y_{2}\right) \end{array}$$

(54) $$\begin{array}{cc} \; & \leq H\left(X^{★}\right)-I\left(X^{★};Y_{2}\right) \end{array}$$

(55) $$\begin{array}{cc} \; & \leq \log(|\mathrm{supp}\left(P_{X^{★}}\right)\left|\right)-I\left(X^{★};Y_{2}\right), \end{array}$$

where the above uses the nonnegativity of the entropy and the fact that entropy is maximized by a uniform distribution. Furthermore, by using a suboptimal uniform (continuous) distribution on [−R,R] as an input and the entropy power inequality, the secrecy capacity is lower-bounded by

(56) $$C_{s}\left(\sigma_{1}^{2},\sigma_{2}^{2},R,1\right)\geq \frac{1}{2}\log\left(1+\frac{\frac{2R^{2}}{\pi e\sigma_{1}^{2}}}{1+\frac{R^{2}}{\sigma_{2}^{2}}}\right).$$

Combining the bounds in (55) and (56), we arrive at the following lower bound on the number of points:

(57) $$|\mathrm{supp}\left(P_{X^{★}}\right)|\geq \sqrt{1+\frac{\frac{2R^{2}}{\pi e\sigma_{1}^{2}}}{1+\frac{R^{2}}{\sigma_{2}^{2}}}}e^{I(X^{★};Y_{2})}.$$

At this point, one needs to determine the behavior of $I(X^{★};Y_{2})$. A trivial lower bound on $\left|\mathrm{supp}(P_{X^{★}})\right|$ can be found by lower-bounding $I(X^{★};Y_{2})$ by zero. However, this lower bound on $\left|\mathrm{supp}(P_{X^{★}})\right|$ does not grow with R, while the upper bound does increase with R. A possible way of establishing a lower bound that increases in R is by showing that $I\left(X^{★};Y_{2}\right)\approx \frac{1}{2}\log\left(1+\frac{R^{2}}{\sigma_{2}^{2}}\right)$. However, because not much is known about the structure of the optimal input distribution $P_{X^{★}}$, it is not immediately evident how one can establish such an approximation or whether it is valid.

## 4. Secrecy Capacity Expression in the Low-Amplitude Regime

The result in Theorem 3 can also be used to establish the secrecy capacity for all $R\leq {\bar{R}}_{n}\left(\sigma_{1}^{2},\sigma_{2}^{2}\right)$, as is performed next.

**Theorem** **6.** *If $G_{\sigma_{1},\sigma_{2},R,n}$ of (39) has at most one sign change and if $R\leq {\bar{R}}_{n}\left(\sigma_{1}^{2},\sigma_{2}^{2}\right)$, then*

(58) $$C_{s}\left(\sigma_{1}^{2},\sigma_{2}^{2},R,n\right)=\frac{1}{2}\int_{\sigma_{1}^{2}}^{\sigma_{2}^{2}}\frac{R^{2}-R^{2}E\left[h_{\frac{n}{2}}^{2}\left(\frac{∥R+\sqrt{s}Z∥R}{s}\right)\right]}{s^{2}}ds.$$

**Proof.**  See Section 9.    □

### Large *n* Asymptotics

It is important to note that as ${\bar{R}}_{n}\left(\sigma_{1}^{2},\sigma_{2}^{2}\right)$ grows as $\sqrt{n}$, according to Theorem 4, when we keep R constant and increase the number of antennas to infinity, the low-amplitude regime becomes the only regime. The next theorem characterizes the secrecy capacity in this ‘massive-MIMO’ regime (i.e., where R is fixed and *n* goes to infinity).

**Theorem** **7.** *Consider the expression in (58) and fix R≥0 and $\sigma_{1}^{2}\leq \sigma_{2}^{2}$, then*

(59) $$\begin{array}{cc} \; & \lim_{n\to \infty }C_{s}\left(\sigma_{1}^{2},\sigma_{2}^{2},R,n\right)=R^{2}\left(\frac{1}{2\sigma_{1}^{2}}-\frac{1}{2\sigma_{2}^{2}}\right). \end{array}$$

**Proof.**  See Appendix C.    □

**Remark** **3.** *The result in Theorem 7 is reminiscent of the capacity in the wideband regime [31, Ch. 9], where the capacity increases linearly in the signal-to-noise ratio. Similarly, Theorem 7 shows that in the large antenna regime, the secrecy capacity grows linearly with the difference in the single-to-noise ratio between the legitimate user and the eavesdropper.*

In Theorem 7, R was held fixed. It is also interesting to study the case when R is a function of *n*. Specifically, it is interesting to study the case when $R=c\sqrt{n}$ for some coefficient *c*.

**Theorem** **8.** *Suppose that $c\leq c(\sigma_{1}^{2},\sigma_{2}^{2})$. Then,*

(60) $$\lim_{n\to \infty }\frac{C_{s}\left(\sigma_{1}^{2},\sigma_{2}^{2},c\sqrt{n},n\right)}{n}=\frac{1}{2}\log\left(\frac{1+c^{2}/\sigma_{1}^{2}}{1+c^{2}/\sigma_{2}^{2}}\right).$$

**Proof.**  See Appendix D.    □

Notice that (60) is equivalent to the secrecy capacity of a vector Gaussian wiretap channel subject to an average power constraint. Gaussian wiretap channels under average power constraints have been extensively investigated [3,32] and, for an average power constraint $E[∥X{∥}^{2}]\leq P$, the resulting secrecy capacity is given by [3]

(61) $$C_{G}\left(\sigma_{1}^{2},\sigma_{2}^{2},P,n\right)=\frac{n}{2}\log\frac{1+P/\sigma_{1}^{2}}{1+P/\sigma_{2}^{2}}.$$

Thus, the result in (60) can be restated as

(62) $$\lim_{n\to \infty }\frac{C_{s}\left(\sigma_{1}^{2},\sigma_{2}^{2},c\sqrt{n},n\right)}{C_{G}\left(\sigma_{1}^{2},\sigma_{2}^{2},c^{2},n\right)}=1.$$

In other words, for the regime considered in Theorem 8, for a large enough *n* the secrecy capacity under the amplitude constraint $R_{n}=c\sqrt{n}$ behaves as the secrecy capacity under the average power constraint $c^{2}$.

## 5. Beyond the Low-Amplitude Regime

To evaluate the secrecy capacity and find the optimal distribution $P_{X^{★}}$ beyond ${\bar{R}}_{n}$ we rely on numerical estimations. We remark that, as pointed out in [25], the secrecy-capacity-achieving distribution is isotropic and consists of finitely many co-centric shells. Keeping this in mind, we can find the optimal input distribution $P_{X^{★}}$ by just optimizing over $P_{∥X∥}$ with ∥X∥≤R.

### 5.1. Numerical Algorithm

In the case of scalar Gaussian wiretap channels, the secrecy capacity and the optimal input pmf can be estimated via the algorithm described in [33], i.e., a numerical procedure that takes inspiration from the deterministic annealing algorithm sketched in [34]. Let us denote by ${\hat{C}}_{s}\left(\sigma_{1}^{2},\sigma_{2}^{2},R,n\right)$ the numerical estimate of the secrecy capacity, and by ${\hat{P}}_{∥X^{★}∥}$, the estimate of the optimal pmf on the input norm. To numerically evaluate ${\hat{C}}_{s}\left(\sigma_{1}^{2},\sigma_{2}^{2},R,n\right)$ and ${\hat{P}}_{∥X^{★}∥}$, we extend to the vector case the algorithm in [33]. Our extension is defined in Algorithm 1. The input parameters of the main function are the noise variances $\sigma_{1}^{2}$ and $\sigma_{2}^{2}$, the radius R, the vectors ρ and p being, respectively, the mass points positions and probabilities of a tentative input pmf, the number of iterations in the while loop $N_{c}$, and finally, a tolerance ε to set the precision of the secrecy capacity estimate.
**Algorithm 1** Secrecy capacity and optimal input pmf estimation1:**procedure** Main$\left(\sigma_{1}^{2},\sigma_{2}^{2},R,\rho ,p,N_{c},\varepsilon \right)$2:    **repeat**3:        k←04:        **while** $k<N_{c}$ **do**5:           k←k+16:           ρ←Gradient Ascent (ρ,p)7:           p←Blahut–Arimoto (ρ,p)8:        **end while**9:        valid ← **KKT** Validation(ρ,p,ε)10:        **if** valid = False **then**11:           (ρ,p)←Add–Point (ρ,p)12:        **end if**13:    **until** valid = True  14:    ${\hat{P}}_{∥X^{★}∥}\leftarrow \left(\rho ,p\right)$  15:    ${\hat{C}}_{s}\left(\sigma_{1}^{2},\sigma_{2}^{2},R,n\right)\leftarrow I_{s}(∥X∥;{\hat{P}}_{∥X^{★}∥})$  16:    **return** ${\hat{P}}_{∥X^{★}∥},{\hat{C}}_{s}\left(\sigma_{1}^{2},\sigma_{2}^{2},R,n\right)$  17:**end procedure**

At its core, the numerical procedure iteratively refines its estimate of $P_{∥X^{★}∥}$ by running a gradient ascent algorithm to update the vector ρ and a variant of the Blahut–Arimoto algorithm [35] to update p.

The Gradient Ascent procedure uses the secrecy information as the objective function and stops either when ρ has reached convergence or at a given maximum number of iterations. Let us denote by $I_{s}(∥X∥;P_{∥X∥})$ the secrecy information as a function of the input norm. Notice that, given a tentative pmf ${\hat{P}}_{∥X∥}$ of mass points ρ, probabilities p, and $|\mathrm{supp}({\hat{P}}_{∥X∥})|=K$, we have

(63) $$\begin{array}{c} I_{s}(∥X∥;{\hat{P}}_{∥X∥})=\sum_{i=1}^{K}p_{i}\cdot \tilde{\Xi }\left(\rho_{i}\;;{\hat{P}}_{∥X∥}\right), \end{array}$$

where $\tilde{\Xi }\left(t;{\hat{P}}_{∥X∥}\right)$ is the secrecy density, with respect to the input norm, defined in (A9) and where $p_{i}$ and $\rho_{i}$ are, respectively, the *i*th element of p and ρ. Then, the Gradient Ascent updates are given by

(64) $$\begin{array}{c} \rho_{i}=\rho_{i}+\alpha \cdot \frac{\partial }{\partial \rho_{i}}I_{s}(∥X∥;{\hat{P}}_{∥X∥}),\;i=1,\cdots ,K, \end{array}$$

where the partial derivatives are defined in Appendix E and α is the step size in the gradient ascent. We remark that, to ensure convergence to a local maximum, we use the gradient ascent algorithm in a backtracking line search version [36]. By suitably adjusting the step size α at each iteration, the backtracking line search version guarantees us that each new update of ρ provides a nondecreasing associated secrecy information, compared to the previous update of ρ.

The Blahut–Arimoto function runs a variant of the Blahut–Arimoto algorithm. For the scalar case, an example of the Blahut–Arimoto optimization, applied to wiretap channels, is given in [37]. Similar results can be extended to the case of vector wiretap channels. Given the current probabilities $p_{i}$’s, the updates are obtained by evaluating

(65) $$\begin{array}{cc} p_{i}^{\prime } & =p_{i}\exp\left(\tilde{\Xi }\left(\rho_{i};{\hat{P}}_{∥X∥}\right)\right),\;i=1,\cdots ,K, \end{array}$$

and finally, by normalizing each $p_{i}^{\prime }$ and assigning them to the entries of the vector p

(66) $$\begin{array}{cc} p_{i} & =\frac{p_{i}^{\prime }}{\sum_{k=1}^{K}p_{i}^{\prime }},\;i=1,\cdots ,K. \end{array}$$

Similarly to Gradient Ascent, the Blahut–Arimoto procedure stops either when the values of p have reached a stable convergence or after a set number of updates.

Since the joint optimization of ρ and p is not numerically feasible, we need to reiterate both the Blahut–Arimoto and the Gradient Ascent procedures a given number of times, namely $N_{c}$. The parameter $N_{c}$ is chosen empirically in such a way that ρ and p become fairly stable, and therefore we can expect to have reached joint convergence for both of them.

Then, the KKT Validation procedure ensures that the values of ρ and p are indeed close to the optimal ones. We check the optimality of ${\hat{P}}_{∥X∥}$ by verifying whether the KKT conditions in Lemma 2 are satisfied. Since the algorithm has to verify the KKT conditions numerically, i.e., with finite precision, we find it more convenient to check the negated version of (28), where a tolerance parameter ε is introduced that trades off accuracy with computational burden. Specifically, ${\hat{P}}_{∥X∥}$ is not an optimal input pmf if any of the following conditions are satisfied:

(67a) $$\begin{array}{cc} \; & |\tilde{\Xi }\left(t;{\hat{P}}_{∥X∥}\right)-I_{s}(∥X∥;{\hat{P}}_{∥X∥})|>\varepsilon ,\;\mathrm{for}\;\mathrm{some}\;t\in \mathrm{supp}\left({\hat{P}}_{∥X∥}\right) \end{array}$$

(67b) $$\begin{array}{cc} \; & I_{s}(∥X∥;{\hat{P}}_{∥X∥})+\varepsilon <\tilde{\Xi }\left(t;{\hat{P}}_{∥X∥}\right),\;\mathrm{for}\;\mathrm{some}\;t\in \left[0,R\right]. \end{array}$$

Note that in (67), in place of the secrecy capacity $C_{s}\left(\sigma_{1}^{2},\sigma_{2}^{2},R,n\right)$, which is unknown, we used the secrecy information given by the tentative pmf ${\hat{P}}_{∥X∥}$, i.e., $I_{s}(∥X∥;{\hat{P}}_{∥X∥})$. Condition (67a) is derived by negating (28a): there exists a $t\in \mathrm{supp}({\hat{P}}_{∥X∥})$, such that $\tilde{\Xi }\left(t;{\hat{P}}_{∥X∥}\right)$ is ε-away from the secrecy information $I_{s}(∥X∥;{\hat{P}}_{∥X∥})$. Condition (67b) is the negated version of (28b): there exists a t∈[0,R] such that $\tilde{\Xi }\left(t;{\hat{P}}_{∥X∥}\right)$ is at least ε-larger than the secrecy information $I_{s}(∥X∥;{\hat{P}}_{∥X∥})$. With some abuse of notation, we refer to (67) as to the ε-KKT conditions. If the tentative pmf ${\hat{P}}_{∥X∥}$ does not pass the check of the ε-KKT conditions, then the algorithm checks whether a new point has to be added to the pmf.

The Add Point procedure evaluates the position of the new mass point

(68) $$\begin{array}{c} \rho_{\mathrm{new}}=\arg\max_{t\in [0,R]}\tilde{\Xi }\left(t;{\hat{P}}_{∥X∥}\right). \end{array}$$

The point $\rho_{\mathrm{new}}$ is appended to the vector ρ and the probabilities p are set to be equiprobable.

The whole procedure is repeated until KKT Validation gives a positive outcome, and at that point the algorithm returns ${\hat{P}}_{∥X^{★}∥}$ as the optimal pmf estimate and ${\hat{C}}_{s}\left(\sigma_{1}^{2},\sigma_{2}^{2},R,n\right)$ as the secrecy capacity estimate.

**Remark** **4.** *In this work, we focus on the secrecy capacity and on the secrecy-capacity-achieving input distribution. However, it is possible to study other points of the rate-equivocation region of the degraded wiretap Gaussian channel by suitably changing the KKT conditions, as reported in [21], Equations (33) and (34). With the due modifications, the proposed optimization algorithm can find the optimal input distribution for any point of the rate-equivocation region.*

### 5.2. Numerical Results

**Figure 2:**
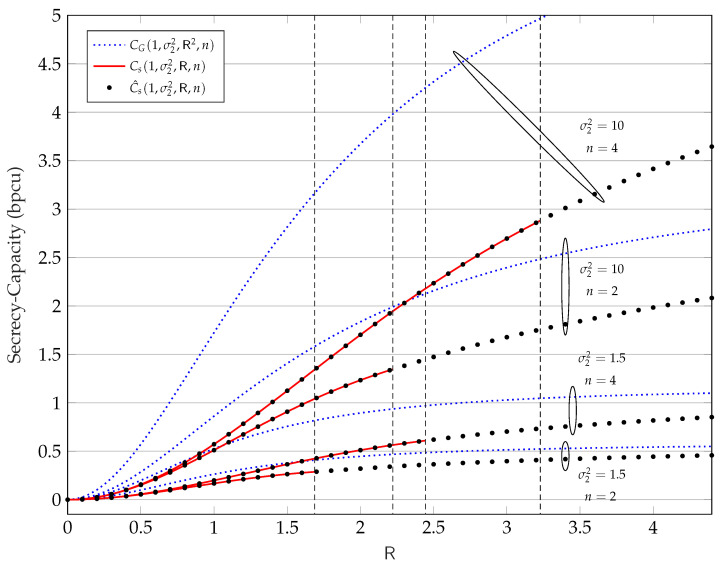
Secrecy capacity in bit per channel use (bpcu) versus R for $\sigma_{2}^{2}=1.5,10$ and n=2,4. The secrecy capacity under average power constraints $C_{G}\left(\sigma_{1}^{2},\sigma_{2}^{2},R^{2},n\right)$ is defined in (69), while under peak power constraints, i.e., $C_{s}\left(\sigma_{1}^{2},\sigma_{2}^{2},R,n\right)$, is defined in (58).

In Figure 2, we show with black dots the numerical estimate ${\hat{C}}_{s}\left(\sigma_{1}^{2},\sigma_{2}^{2},R,n\right)$ versus R, evaluated via Algorithm 1, for $\sigma_{1}^{2}=1$, $\sigma_{2}^{2}=1.5,10$, n=2,4, and tolerance $\varepsilon =10^{-6}$. For the same values of $\sigma_{1}^{2}$, $\sigma_{2}^{2}$, and *n* we also show, with the red lines, the analytical low-amplitude regime secrecy capacity $C_{s}\left(\sigma_{1}^{2},\sigma_{2}^{2},R,n\right)$ versus R from Theorem 6. In addition, we show with blue dotted lines the secrecy capacity under the average power constraint $E\left[{∥X∥}^{2}\right]\leq R^{2}$:

(69) $$\begin{array}{cc} C_{G}\left(\sigma_{1}^{2},\sigma_{2}^{2},R^{2},n\right) & =\frac{n}{2}\log\frac{1+R^{2}/\sigma_{1}^{2}}{1+R^{2}/\sigma_{2}^{2}}\geq C_{s}\left(\sigma_{1}^{2},\sigma_{2}^{2},R,n\right), \end{array}$$

where the inequality follows by noting that the average power constraint $E\left[{∥X∥}^{2}\right]\leq R^{2}$ is weaker than the amplitude constraint ∥X∥≤R. Finally, the dashed vertical lines show ${\bar{R}}_{n}$, i.e., the upper limit of the low-amplitude regime, for the considered values of $\sigma_{1}^{2}$, $\sigma_{2}^{2}$, and *n*.

**Figure 3:**
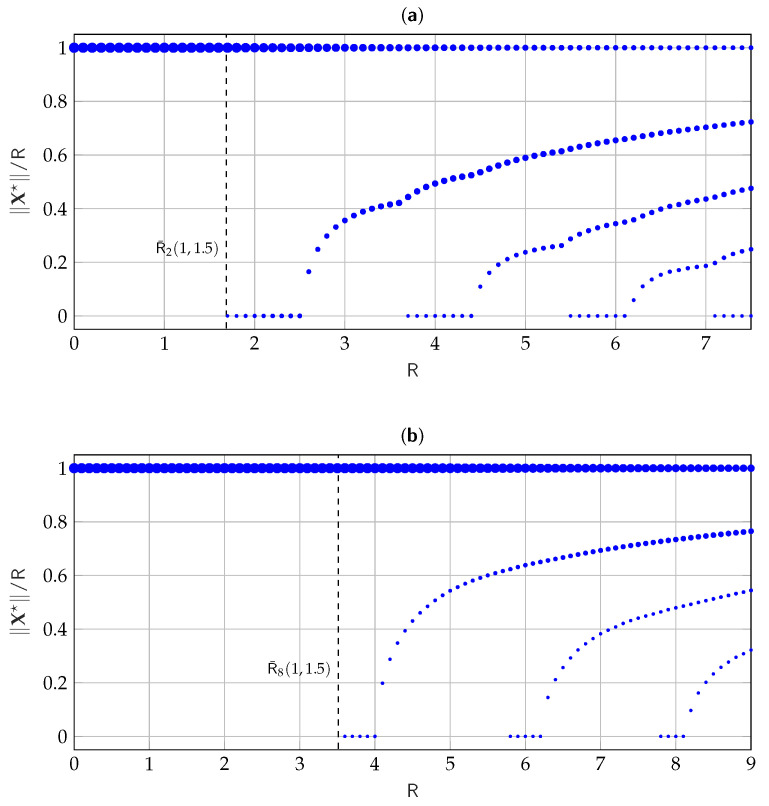
Evolution of the numerically estimated ${\hat{P}}_{∥X^{★}∥}$ versus R for $\sigma_{1}^{2}=1$, $\sigma_{2}^{2}=1.5$, (**a**) n=2, and (**b**) n=8.

**Figure 4:**
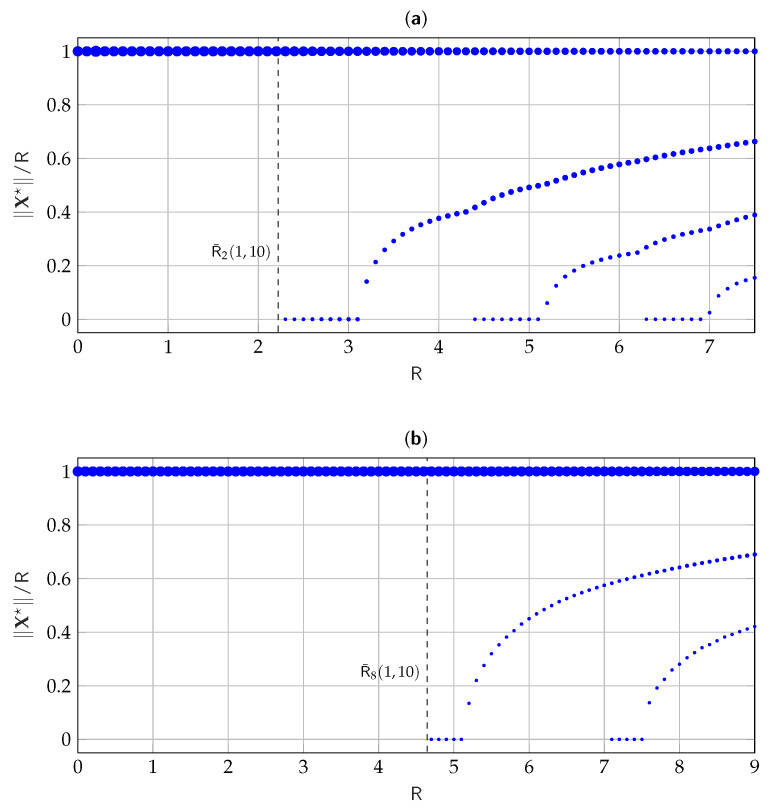
Evolution of the numerically estimated ${\hat{P}}_{∥X^{★}∥}$ versus R for $\sigma_{1}^{2}=1$, $\sigma_{2}^{2}=10$, (**a**) n=2, and (**b**) n=8.

In Figure 3, we consider discrete values for R and for each value of R we plot the corresponding estimated pmf ${\hat{P}}_{∥X^{★}∥}$, evaluated via Algorithm 1, for $\sigma_{1}^{2}=1$, $\sigma_{2}^{2}=1.5$, n=2,8, and tolerance $\varepsilon =10^{-6}$. The figure shows, at each R, the normalized amplitude of support points in the estimated pmf, while the size of the circles qualitatively shows the probability associated with each support point. Similarly, Figure 4 shows the evolution of the pmf estimate for $\sigma_{1}^{2}=1$, $\sigma_{2}^{2}=10$, n=2,8, and $\varepsilon =10^{-6}$. It is interesting to notice how in both Figure 3 and Figure 4 when a new mass point is added to the pmf, it appears in zero. Moreover, the mass point of radius R always seems to be optimal.

**Figure 5:**
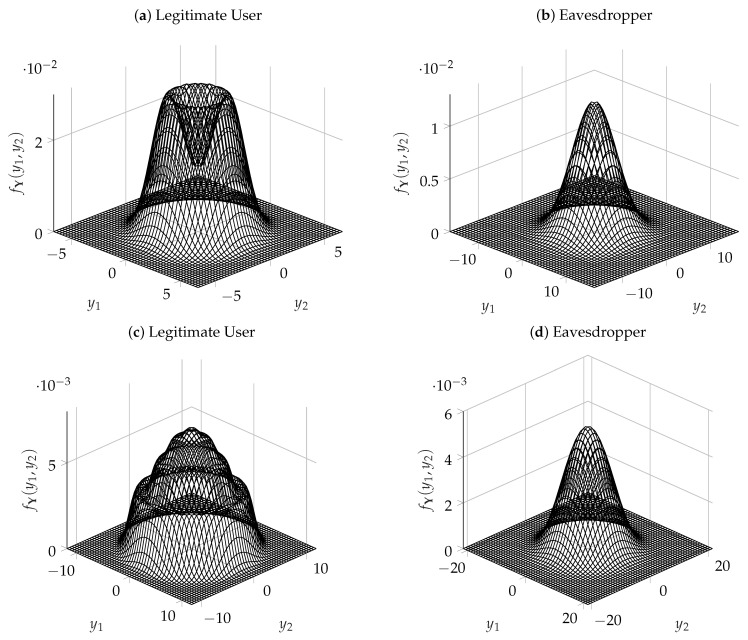
Output pdf of the legitimate user and of the eavesdropper for $\sigma_{1}^{2}=1$, $\sigma_{2}^{2}=10$, n=2, (**a**,**b**) R=2.25, and (**c**,**d**) R=7.5. An animation showing the evolution of the output pdf as R varies can be found in [1].

Finally, Figure 5 shows the output distributions of the legitimate user and of the eavesdropper in the case of $\sigma_{1}^{2}=1$, $\sigma_{2}^{2}=10$, n=2, and for two values of R. At the top of the figure, the distributions are shown for R=2.25, which is a value close to ${\bar{R}}_{2}\left(1,10\right)$. At the bottom of the figure, the distributions are shown for R=7.5. For both values of R, the legitimate user sees an output distribution where the co-centric rings of the input distribution are easily distinguishable. On the other hand, as expected, the output distribution seen by the eavesdropper is close to a Gaussian.

## 6. Proof of Theorem 3

### Estimation Theoretic Representation

By Remark 1, if $G_{\sigma_{1},\sigma_{2},R,n}$ has at most one sign change, $P_{X_{R}}$ is secrecy-capacity-achieving if, and only if, for all ∥x∥=R

(70) $$\Xi \left(0;P_{X_{R}}\right)\leq \Xi \left(x;P_{X_{R}}\right).$$

We seek to re-write the condition (70) in the estimation theoretic form. To that end, we need the following representation of the relative entropy [38]:

(71) $$D\left(P_{X_{1}+\sqrt{t}Z}∥P_{X_{2}+\sqrt{t}Z}\right)=\frac{1}{2}\int_{t}^{\infty }\frac{g(s)}{s^{2}}ds,$$

where

(72) $$\begin{array}{cc} g(s) & =E\left[∥X_{1}-\ell_{2}\left(X_{1}+\sqrt{s}Z\right){∥}^{2}\right]-E\left[∥X_{1}-\ell_{1}\left(X_{1}+\sqrt{s}Z\right){∥}^{2}\right] \end{array}$$

and where

(73) $$\begin{array}{cc} \ell_{i}\left(y\right) & =E[X_{i}|X_{i}+\sqrt{s}Z=y] \end{array}$$

(74) $$\begin{array}{cc} \; & =\int x_{i}f_{X_{i}|X_{i}+\sqrt{s}Z}\left(x_{i}|y\right)dx_{i},\;i\in \left\{1,2\right\}. \end{array}$$

Another fact that will be important for our expression is

(75) $$\begin{array}{c} E\left[X_{R}|X_{R}+\sqrt{s}Z=y\right]=\frac{Ry}{∥y∥}h_{\frac{n}{2}}\left(\frac{∥y∥R}{s}\right), \end{array}$$

see, for example [27], for the proof.

Next, using (71) and (75) note that for any ∥x∥=R we have that for i∈{1,2}

(76) $$\begin{array}{cc} D(P_{x+\sqrt{\sigma_{i}^{2}}Z}∥P_{X_{R}+\sqrt{\sigma_{i}^{2}}Z})\; & =\frac{1}{2}\int_{\sigma_{i}^{2}}^{\infty }\frac{E\left[{∥x-\frac{R(x+\sqrt{s}Z)}{∥x+\sqrt{s}Z∥}h_{\frac{n}{2}}\left(\frac{∥x+\sqrt{s}Z∥R}{s}\right)∥}^{2}\right]}{s^{2}}ds \end{array}$$

(77) $$\begin{array}{cc} \; & =\frac{1}{2}\int_{\sigma_{i}^{2}}^{\infty }\frac{E\left[{∥x∥}^{2}\right]-E\left[{∥\frac{R(x+\sqrt{s}Z)}{∥x+\sqrt{s}Z∥}h_{\frac{n}{2}}\left(\frac{∥x+\sqrt{s}Z∥R}{s}\right)∥}^{2}\right]}{s^{2}}ds \end{array}$$

(78) $$\begin{array}{cc} \; & =\frac{1}{2}\int_{\sigma_{i}^{2}}^{\infty }\frac{R^{2}-R^{2}E\left[h_{\frac{n}{2}}^{2}\left(\frac{∥x+\sqrt{s}Z∥R}{s}\right)\right]}{s^{2}}ds, \end{array}$$

where (77) follows from

(79) $$\begin{array}{cc} \mathrm{mmse}(X_{R}|Y) & =E\left[∥X_{R}-E\left[X_{R}|Y\right]{∥}^{2}\right] \end{array}$$

(80) $$\begin{array}{cc} \; & =E\left[∥X_{R}{∥}^{2}\right]-E\left[∥E\left[X_{R}|Y\right]{∥}^{2}\right]. \end{array}$$

Moreover, for ∥x∥=0, it holds

(81) $$\begin{array}{c} D\left(P_{0+\sqrt{\sigma_{i}^{2}}Z}∥P_{X_{R}+\sqrt{\sigma_{i}^{2}}Z}\right)=\frac{1}{2}\int_{\sigma_{i}^{2}}^{\infty }\frac{R^{2}E\left[h_{\frac{n}{2}}^{2}\left(\frac{R∥Z∥}{s}\right)\right]}{s^{2}}ds. \end{array}$$

Now, note that by using the definition of $\Xi (x;P_{X_{R}})$ in (30), (78), and (81) we have that for ∥x∥=R

(82) $$\begin{array}{cc} \Xi (x;P_{X_{R}}) & =D\left(P_{x+\sqrt{\sigma_{1}^{2}}Z}∥P_{X_{R}+\sqrt{\sigma_{1}^{2}}Z}\right)-D\left(P_{x+\sqrt{\sigma_{2}^{2}}Z}∥P_{X_{R}+\sqrt{\sigma_{2}^{2}}Z}\right) \end{array}$$

(83) $$\begin{array}{cc} \; & =\frac{1}{2}\int_{\sigma_{1}^{2}}^{\sigma_{2}^{2}}\frac{R^{2}-R^{2}E\left[h_{\frac{n}{2}}^{2}\left(\frac{∥x+\sqrt{s}Z∥R}{s}\right)\right]}{s^{2}}ds, \end{array}$$

and

(84) $$\begin{array}{cc} \Xi (0;P_{X_{R}}) & =D\left(P_{0+\sqrt{\sigma_{1}^{2}}Z}∥P_{X_{R}+\sqrt{\sigma_{1}^{2}}Z}\right)-D\left(P_{0+\sqrt{\sigma_{2}^{2}}Z}∥P_{X_{R}+\sqrt{\sigma_{2}^{2}}Z}\right) \end{array}$$

(85) $$\begin{array}{cc} \; & =\frac{1}{2}\int_{\sigma_{1}^{2}}^{\sigma_{2}^{2}}\frac{R^{2}E\left[h_{\frac{n}{2}}^{2}\left(\frac{∥\sqrt{s}Z∥R}{s}\right)\right]}{s^{2}}ds \end{array}$$

Consequently, the necessary and sufficient condition in Theorem 2 can be equivalently written as

(86) $$\begin{array}{cc} \; & \int_{\sigma_{1}^{2}}^{\sigma_{2}^{2}}\frac{E\left[h_{\frac{n}{2}}^{2}\left(\frac{∥\sqrt{s}Z∥R}{s}\right)+h_{\frac{n}{2}}^{2}\left(\frac{∥x+\sqrt{s}Z∥R}{s}\right)\right]-1}{s^{2}}ds\leq 0. \end{array}$$

Now ${\bar{R}}_{n}\left(\sigma_{1}^{2},\sigma_{2}^{2}\right)$ will be the largest R that satisfies (86), which concludes the proof of Theorem 3.

## 7. Proof of Theorem 4

The objective of the proof is to understand how the condition in (45) behaves as n→∞. To study the large *n* behavior, we need to the following bounds on the $h_{\nu }$ [39,40]: for $\nu >\frac{1}{2}$

(87) $$\begin{array}{c} h_{\nu }\left(x\right)=\frac{x}{\frac{2\nu -1}{2}+\sqrt{\frac{{\left(2\nu -1\right)}^{2}}{4}+x^{2}}}\cdot g_{\nu }\left(x\right), \end{array}$$

where

(88) $$\begin{array}{c} 1\geq g_{\nu }\left(x\right)\geq \frac{\frac{2\nu -1}{2}+\sqrt{\frac{{\left(2\nu -1\right)}^{2}}{4}+x^{2}}}{\nu +\sqrt{\nu^{2}+x^{2}}}. \end{array}$$

Now let $R=c\sqrt{n}$ for some c>0. The goal is to understand the behavior of

(89) $$E\left[h_{\frac{n}{2}}^{2}\left(\frac{∥\sqrt{s}Z∥R}{s}\right)+h_{\frac{n}{2}}^{2}\left(\frac{∥x+\sqrt{s}Z∥R}{s}\right)\right]$$

as *n* goes to infinity. First, let

(90) $$\begin{array}{c} V_{n}=\frac{∥Z∥}{\sqrt{n}}, \end{array}$$

and note that

(91) $$\begin{array}{cc} \lim_{n\to \infty }E\left[h_{\frac{n}{2}}^{2}\left(\frac{∥\sqrt{s}Z∥c\sqrt{n}}{s}\right)\right]\; & =\lim_{n\to \infty }E\left[{\left(\frac{\frac{cV_{n}}{\sqrt{s}}}{\frac{n-1}{2n}+\sqrt{\frac{{\left(n-1\right)}^{2}}{4n^{2}}+{\left(\frac{cV_{n}}{\sqrt{s}}\right)}^{2}}}\cdot g_{\frac{n}{2}}\left(\frac{cV_{n}}{\sqrt{s}}n\right)\right)}^{2}\right] \end{array}$$

(92) $$\begin{array}{cc} \; & =E\left[\lim_{n\to \infty }{\left(\frac{\frac{cV_{n}}{\sqrt{s}}}{\frac{n-1}{2n}+\sqrt{\frac{{\left(n-1\right)}^{2}}{4n^{2}}+{\left(\frac{cV_{n}}{\sqrt{s}}\right)}^{2}}}\cdot g_{\frac{n}{2}}\left(\frac{cV_{n}}{\sqrt{s}}n\right)\right)}^{2}\right] \end{array}$$

(93) $$\begin{array}{cc} \; & =\frac{c^{2}}{{\left(\frac{\sqrt{s}}{2}+\sqrt{\frac{s}{4}+c^{2}}\right)}^{2}}, \end{array}$$

where (92) follows from the dominated convergence theorem, and (93) follows since, by the law of large numbers we have, almost surely,

(94) $$\begin{array}{c} \lim_{n\to \infty }V_{n}^{2}=\lim_{n\to \infty }\frac{1}{n}\sum_{i=1}^{n}Z_{i}^{2}=E\left[Z^{2}\right]=1. \end{array}$$

Second, let

(95) $$\begin{array}{c} W_{n}=\frac{∥x+\sqrt{s}Z∥}{\sqrt{n}}, \end{array}$$

where, without loss of generality, we take x=[R,0,…,0]

(96) $$\begin{array}{cc} \lim_{n\to \infty }E\left[h_{\frac{n}{2}}^{2}\left(\frac{∥x+\sqrt{s}Z∥c\sqrt{n}}{s}\right)\right]\; & =\lim_{n\to \infty }E\left[{\left(\frac{\frac{cW_{n}}{s}\cdot g_{\frac{n}{2}}\left(\frac{cW_{n}}{s}n\right)}{\frac{n-1}{2n}+\sqrt{\frac{{\left(n-1\right)}^{2}}{4n^{2}}+{\left(\frac{cW_{n}}{s}\right)}^{2}}}\right)}^{2}\right] \end{array}$$

(97) $$\begin{array}{cc} \; & =E\left[\lim_{n\to \infty }{\left(\frac{\frac{cW_{n}}{s}\cdot g_{\frac{n}{2}}\left(\frac{cW_{n}}{s}n\right)}{\frac{n-1}{2n}+\sqrt{\frac{{\left(n-1\right)}^{2}}{4n^{2}}+{\left(\frac{cW_{n}}{s}\right)}^{2}}}\right)}^{2}\right] \end{array}$$

(98) $$\begin{array}{cc} \; & =\frac{c^{2}\left(c^{2}+s\right)}{{\left(\frac{s}{2}+\sqrt{\frac{s^{2}}{4}+c^{2}\left(c^{2}+s\right)}\right)}^{2}}, \end{array}$$

where (97) follows from the dominated convergence theorem and where (98) follows since, by the strong law of large numbers we have, almost surely,

(99) $$\begin{array}{cc} \lim_{n\to \infty }W_{n}^{2} & =\lim_{n\to \infty }\frac{1}{n}{\left(\sqrt{s}Z_{1}+c\sqrt{n}\right)}^{2}+s\lim_{n\to \infty }\frac{1}{n}\sum_{i=2}^{n}Z_{i}^{2} \end{array}$$

(100) $$\begin{array}{cc} \; & =c^{2}+s. \end{array}$$

Combining (93) and (98) with (45), we arrive at

(101) $$\begin{array}{cc} \; & \int_{\sigma_{1}^{2}}^{\sigma_{2}^{2}}\frac{\frac{c^{2}}{{\left(\frac{\sqrt{s}}{2}+\sqrt{\frac{s}{4}+c^{2}}\right)}^{2}}+\frac{c^{2}\left(c^{2}+s\right)}{{\left(\frac{s}{2}+\sqrt{\frac{s^{2}}{4}+c^{2}\left(c^{2}+s\right)}\right)}^{2}}-1}{s^{2}}ds=0. \end{array}$$

## 8. Proof of Theorem 5

### 8.1. Implicit Upper Bound

A consequence of the KKT conditions of Lemma 1 is the inclusion

(102) $$\mathrm{supp}\left(P_{X^{★}}\right)\subseteq \left\{x\in \left[-R,R\right]:\Xi \left(x\right)-C_{s}=0\right\}$$

which suggests the following upper bound on the number of support points of $P_{X^{★}}$:

(103) $$\begin{array}{cc} \left|\mathrm{supp}(P_{X^{★}})\right| & \leq N\left(\left[-R,R\right],\Xi \left(x\right)-C_{s}\left(\sigma_{1}^{2},\sigma_{2}^{2},R,1\right)\right) \end{array}$$

(104) $$\begin{array}{cc} \; & =N\left(\left[-R,R\right],E\left[g\left(Y_{1}\right)+\log\left(\frac{\sigma_{2}}{\sigma_{1}}\right)-C_{s}|X=x\right]\right) \end{array}$$

(105) $$\begin{array}{cc} \; & \leq S\left(g\left(\cdot \right)+\log\left(\frac{\sigma_{2}}{\sigma_{1}}\right)-C_{s}\right) \end{array}$$

(106) $$\begin{array}{cc} \; & \leq N\left(R,g\left(\cdot \right)+\log\left(\frac{\sigma_{2}}{\sigma_{1}}\right)-C_{s}\right) \end{array}$$

(107) $$\begin{array}{cc} \; & =N\left(\left[-L,L\right],g\left(\cdot \right)+\log\left(\frac{\sigma_{2}}{\sigma_{1}}\right)-C_{s}\right) \end{array}$$

(108) $$\begin{array}{cc} \; & <\infty , \end{array}$$

where (104) follows from using (21); (105) follows from applying Karlin’s oscillation Theorem 1 and the fact that the Gaussian pdf is a strictly totally positive kernel, which was shown in [26]; (107) is proved in Lemma A3 in the Appendix B; and (108) follows because g(·) is an analytic function in (−L,L). The implicit upper bound (49) of Theorem 5 follows from (107) and (108).

### 8.2. Explicit Upper Bound

The key to finding an explicit upper bound on the number of zeros will be the following complex-analytic result.

**Lemma** **3** (Tijdeman’s Number of Zeros Lemma [41]). *Let L,s,t be positive numbers, such that s>1. For the complex valued function f≠0, which is analytic on |z|<(st+s+t)L, its number of zeros $N(D_{L},f)$ within the disk $D_{L}=\left\{z:|z|\leq L\right\}$ satisfies*

(109) $$\begin{array}{cc} N(D_{L},f) & \leq \frac{1}{\log s}\left(\log\max_{|z|\leq (st+s+t)L}\left|f\left(z\right)\right|-\log\max_{|z|\leq tL}\left|f\left(z\right)\right|\right). \end{array}$$

Furthermore, the following loosened version of the implicit upper bound in (49) will be useful.

**Lemma** **4.**
(110) $$\begin{array}{cc} |\mathrm{supp}(P_{X^{★}})|\; & \leq N\left([-L,L],h(\cdot )\right)+1 \end{array}$$

*where*

(111) $$\begin{array}{cc} \frac{h(y)}{\sigma_{1}^{2}f_{Y_{1}}\left(y\right)} & =\frac{E_{N}\left[E[X^{★}|Y_{2}=y+N]\right]-y}{\sigma_{2}^{2}}-\frac{E[X^{★}|Y_{1}=y]-y}{\sigma_{1}^{2}} \end{array}$$

(112) $$\begin{array}{cc} \; & =\frac{E\left[N\log f_{Y_{2}}\left(y+N\right)\right]}{\sigma_{2}^{2}-\sigma_{1}^{2}}-\frac{E[X^{★}|Y_{1}=y]-y}{\sigma_{1}^{2}}, \end{array}$$

*and where $N∼N(0,\sigma_{2}^{2}-\sigma_{1}^{2})$.*

**Proof.**  Starting from (107), we can write

(113) $$\begin{array}{cc} \left|\mathrm{supp}(P_{X^{★}})\right| & \leq N\left(\left[-L,L\right],g\left(\cdot \right)+\log\left(\frac{\sigma_{2}}{\sigma_{1}}\right)-C_{s}\right) \end{array}$$

(114) $$\begin{array}{cc} \; & \leq N\left(\left[-L,L\right],g^{\prime }\left(\cdot \right)\right)+1 \end{array}$$

(115) $$\begin{array}{cc} \; & =N\left(\left[-L,L\right],\sigma_{1}^{2}f_{Y_{1}}\left(\cdot \right)g^{\prime }\left(\cdot \right)\right)+1 \end{array}$$

where in step (114), we applied Rolle’s theorem, and in step (115), we used the fact that multiplying by a strictly positive function (i.e., $\sigma_{1}^{2}f_{Y_{1}}$) does not change the number of zeros. The first derivative of *g* can be computed as follows:

(116) $$\begin{array}{cc} g^{\prime }\left(y\right) & =E\left[\frac{d}{dy}\log f_{Y_{2}}\left(y+N\right)\right]-\frac{d}{dy}\log f_{Y_{1}}\left(y\right) \end{array}$$

(117) $$\begin{array}{cc} \; & =\frac{E_{N}\left[E[X^{★}|Y_{2}=y+N]\right]-y}{\sigma_{2}^{2}}-\frac{E[X^{★}|Y_{1}=y]-y}{\sigma_{1}^{2}}, \end{array}$$

where in the last step, we used the well-known Tweedie’s formula (see for example [42,43]):

(118) $$E\left[X^{★}|Y_{i}=y\right]=y+\sigma_{i}^{2}\frac{d}{dy}\log f_{Y_{i}}\left(y\right).$$

An alternative expression for the first term in the right-hand side (RHS) of (116) is as follows:

(119) $$\begin{array}{cc} E\left[\frac{d}{dy}\log f_{Y_{2}}\left(y+N\right)\right] & =\int_{-\infty }^{\infty }f_{N}\left(n\right)\frac{d}{dy}\log f_{Y_{2}}\left(y+n\right)dn \end{array}$$

(120) $$\begin{array}{cc} \; & =-\int_{-\infty }^{\infty }\left(\frac{d}{dn}f_{N}\left(n\right)\right)\cdot \log f_{Y_{2}}\left(y+n\right)dn \end{array}$$

(121) $$\begin{array}{cc} \; & =\int_{-\infty }^{\infty }\frac{n}{\sigma_{2}^{2}-\sigma_{1}^{2}}f_{N}\left(n\right)\cdot \log f_{Y_{2}}\left(y+n\right)dn \end{array}$$

(122) $$\begin{array}{cc} \; & =\frac{1}{\sigma_{2}^{2}-\sigma_{1}^{2}}E\left[N\log f_{Y_{2}}\left(y+N\right)\right], \end{array}$$

where $f_{N}\left(n\right)=\phi_{\sqrt{\sigma_{2}^{2}-\sigma_{1}^{2}}}\left(n\right)$. The proof is concluded by letting

(123) $$\begin{array}{c} h\left(y\right)≜\sigma_{1}^{2}f_{Y_{1}}\left(y\right)g^{\prime }\left(y\right). \end{array}$$

□

To apply Tijdeman’s number of zeros Lemma, upper and lower bounds to the maximum module of the complex analytic extension of *h* over the disk $D_{L}=\left\{z:|z|\leq L\right\}$ are proposed in Lemmas A4 and A5 in the Appendix B. Using those bounds, we can provide an upper bound on the number of mass points as follows:

$$\begin{array}{cc} \; & N\left([-L,L],h(\cdot )\right) \end{array}$$

(124) $$\begin{array}{c} \;\leq N\left(D_{L},\overset{˘}{h}\left(\cdot \right)\right) \end{array}$$

(125) $$\begin{array}{cc} \; & \;\leq \min_{s>1,\;t>0}\left\{\frac{\log\frac{\max_{|z|\leq (st+s+t)L}\left|\overset{˘}{h}\left(z\right)\right|}{\max_{|z|\leq tL}\left|\overset{˘}{h}\left(z\right)\right|}}{\log s}\right\} \end{array}$$

(126) $$\begin{array}{cc} \; & \;\leq \log\frac{\frac{e^{\frac{{\left(2e+1\right)}^{2}L^{2}}{2\sigma_{1}^{2}}}}{\sqrt{2\pi \sigma_{1}^{2}}}\left(a_{1}{\left(2e+1\right)}^{2}L^{2}+a_{2}\left(2e+1\right)L+a_{3}\right)}{\left(c_{1}L-c_{2}R\right)\frac{\exp\left(-\frac{{\left(L+R\right)}^{2}}{2\sigma_{1}^{2}}\;\right)}{\sqrt{2\pi \sigma_{1}^{2}}}} \end{array}$$

(127) $$\begin{array}{cc} \; & \;=\frac{{\left(2e+1\right)}^{2}L^{2}}{2\sigma_{1}^{2}}+\frac{{\left(L+R\right)}^{2}}{2\sigma_{1}^{2}}+\log\frac{a_{1}{\left(2e+1\right)}^{2}L^{2}+a_{2}\left(2e+1\right)L+a_{3}}{c_{1}L-c_{2}R}\\ \; & \;=\frac{{\left(2e+1\right)}^{2}{\left(d_{1}R+d_{2}\right)}^{2}}{2\sigma_{1}^{2}}+\frac{{\left(\left(d_{1}+1\right)R+d_{2}\right)}^{2}}{2\sigma_{1}^{2}} \end{array}$$

(128) $$\begin{array}{cc} \; & \;+\log\frac{a_{1}{\left(2e+1\right)}^{2}{\left(d_{1}R+d_{2}\right)}^{2}+a_{2}\left(2e+1\right)\left(d_{1}R+d_{2}\right)+a_{3}}{\left(c_{1}d_{1}-c_{2}\right)R+c_{1}d_{2}} \end{array}$$

(129) $$\begin{array}{cc} \; & \;\leq b_{1}\frac{R^{2}}{\sigma_{1}^{2}}+b_{2}+\log\frac{b_{3}R^{2}+b_{4}R+b_{5}}{b_{6}R+b_{7}} \end{array}$$

(130) $$\begin{array}{cc} \; & \;\leq b_{1}\frac{R^{2}}{\sigma_{1}^{2}}+O\left(\log\left(R\right)\right), \end{array}$$

where (124) follows because extending to a larger domain can only increase the number of zeros; (125) follows from the Tijdeman’s Number of Zeros Lemma; (126) follows from choosing s=e and t=1 and using bounds in Lemmas A4 and A5; (128) follows from using the value of *L* in (A38); (129) using the bound ${\left(a+b\right)}^{2}\leq 2\left(a^{2}+b^{2}\right)$ and defining

(131a) $$\begin{array}{cc} b_{1} & ={\left(2e+1\right)}^{2}d_{1}^{2}+{\left(d_{1}+1\right)}^{2} \end{array}$$

(131b) $$\begin{array}{cc} \; & ={\left(2e+1\right)}^{2}{\left(\frac{\sigma_{2}+\sigma_{1}}{\sigma_{2}-\sigma_{1}}\right)}^{2}+{\left(\frac{\sigma_{2}+\sigma_{1}}{\sigma_{2}-\sigma_{1}}+1\right)}^{2} \end{array}$$

(131c) $$\begin{array}{cc} b_{2} & =\frac{\left({\left(2e+1\right)}^{2}+1\right)d_{2}^{2}}{\sigma_{1}^{2}} \end{array}$$

(131d) $$\begin{array}{cc} \; & =\frac{\left({\left(2e+1\right)}^{2}+1\right)}{\sigma_{1}^{2}}\frac{\frac{\sigma_{2}^{2}-\sigma_{1}^{2}}{\sigma_{2}^{2}}+2C_{s}}{\frac{1}{\sigma_{1}^{2}}-\frac{1}{\sigma_{2}^{2}}} \end{array}$$

(131e) $$\begin{array}{cc} \; & =\left({\left(2e+1\right)}^{2}+1\right)\left(1+2\frac{\sigma_{2}^{2}}{\sigma_{2}^{2}-\sigma_{1}^{2}}C_{s}\right) \end{array}$$

(131f) $$\begin{array}{cc} b_{3} & =2{\left(2e+1\right)}^{2}a_{1}d_{1}^{2} \end{array}$$

(131g) $$\begin{array}{cc} \; & =2{\left(2e+1\right)}^{2}\frac{3\sigma_{1}^{2}}{\sigma_{2}^{2}\sqrt{\sigma_{2}^{2}-\sigma_{1}^{2}}}{\left(\frac{\sigma_{2}+\sigma_{1}}{\sigma_{2}-\sigma_{1}}\right)}^{2} \end{array}$$

(131h) $$\begin{array}{cc} b_{4} & =\left(2e+1\right)d_{1}a_{2} \end{array}$$

(131i) $$\begin{array}{cc} \; & =\left(2e+1\right)\frac{\sigma_{2}+\sigma_{1}}{\sigma_{2}-\sigma_{1}}\left(\frac{\sqrt{2}\sigma_{1}^{2}}{\sqrt{\sigma_{2}^{2}}\sqrt{\sigma_{2}^{2}-\sigma_{1}^{2}}}+2\right) \end{array}$$

(131j) $$\begin{array}{cc} b_{5} & =2{\left(2e+1\right)}^{2}a_{1}d_{2}^{2}+\left(2e+1\right)a_{2}d_{2}+a_{3}\\ \; & =2{\left(2e+1\right)}^{2}\frac{3\sigma_{1}^{2}}{\sigma_{2}^{2}\sqrt{\sigma_{2}^{2}-\sigma_{1}^{2}}}\left(\frac{\frac{\sigma_{2}^{2}-\sigma_{1}^{2}}{\sigma_{2}^{2}}+2C_{s}}{\frac{1}{\sigma_{1}^{2}}-\frac{1}{\sigma_{2}^{2}}}\right)\\ \; & +\left(2e+1\right)\left(\frac{\sqrt{2}\sigma_{1}^{2}}{\sqrt{\sigma_{2}^{2}}\sqrt{\sigma_{2}^{2}-\sigma_{1}^{2}}}+2\right)\sqrt{\frac{\frac{\sigma_{2}^{2}-\sigma_{1}^{2}}{\sigma_{2}^{2}}+2C_{s}}{\frac{1}{\sigma_{1}^{2}}-\frac{1}{\sigma_{2}^{2}}}} \end{array}$$

(131k) $$\begin{array}{cc} \; & +\frac{\sigma_{1}^{2}}{\sqrt{\sigma_{2}^{2}-\sigma_{1}^{2}}}\cdot \sqrt{|\log\left(2\pi \sigma_{2}^{2}\right){|}^{2}+\frac{24{\left(\sigma_{2}^{2}-\sigma_{1}^{2}\right)}^{2}}{\sigma_{2}^{4}}+\pi^{2}} \end{array}$$

(131l) $$\begin{array}{cc} b_{6} & =c_{1}d_{1}-c_{2} \end{array}$$

(131m) $$\begin{array}{cc} \; & =\frac{\sigma_{2}^{2}-\sigma_{1}^{2}}{\sigma_{2}^{2}}\frac{\sigma_{2}+\sigma_{1}}{\sigma_{2}-\sigma_{1}}-\frac{\sigma_{2}^{2}+\sigma_{1}^{2}}{\sigma_{2}^{2}}=2\frac{\sigma_{1}}{\sigma_{2}} \end{array}$$

(131n) $$\begin{array}{cc} b_{7} & =c_{1}d_{2} \end{array}$$

(131o) $$\begin{array}{cc} \; & =\frac{\sigma_{2}^{2}-\sigma_{1}^{2}}{\sigma_{2}^{2}}\sqrt{\frac{\frac{\sigma_{2}^{2}-\sigma_{1}^{2}}{\sigma_{2}^{2}}+2C_{s}}{\frac{1}{\sigma_{1}^{2}}-\frac{1}{\sigma_{2}^{2}}}}; \end{array}$$

and (130) follows from the fact that the $b_{1},b_{3},b_{4}$, and $b_{6}$ coefficients do not depend on R and the fact that the coefficients $b_{2},b_{5}$, and $b_{4}$, while they do depend on R through $C_{s}$, do not grow with R. The fact that $C_{s}$ does not grow with R follows from the bound in (69).

Finally, the explicit upper bound on the number of support points of $P_{X^{★}}$ in (52) is a consequence of (130).

## 9. Proof of Theorem 6

Using the KKT conditions in (28), we have that for x=[R,0,…,0]

(132) $$\begin{array}{cc} C_{s}\left(\sigma_{1}^{2},\sigma_{2}^{2},R,n\right) & =\Xi (x;P_{X_{R}}) \end{array}$$

(133) $$\begin{array}{cc} \; & =D\left(f_{Y_{1}|X}\left(\cdot |x\right)∥f_{Y_{1}^{★}}\right)-D\left(f_{Y_{2}|X}\left(\cdot |x\right)∥f_{Y_{2}^{★}}\right) \end{array}$$

(134) $$\begin{array}{cc} \; & =\frac{1}{2}\int_{\sigma_{1}^{2}}^{\sigma_{2}^{2}}\frac{R^{2}-R^{2}E\left[h_{\frac{n}{2}}^{2}\left(\frac{∥R+\sqrt{s}Z∥R}{s}\right)\right]}{s^{2}}ds \end{array}$$

where the last expression was computed in (83). This concludes the proof.

## 10. Conclusions

This paper has focused on the secrecy capacity of the *n*-dimensional vector Gaussian wiretap channel under the peak power (or amplitude constraint) in a so-called low (but not vanishing) amplitude regime. In this regime, the optimal input distribution $P_{X_{R}}$ is supported on a single *n*-dimensional sphere of radius R. The paper has identified the largest ${\bar{R}}_{n}$, such that the distribution $P_{X_{R}}$ is optimal. In addition, the asymptotic of ${\bar{R}}_{n}$ has been completely characterized as dimension *n* approaches infinity. As a by-product of the analysis, the capacity in the low-amplitude regime has also been characterized in a more or less closed form. The paper has also provided a number of supporting numerical examples. Implicit and explicit upper bounds have been proposed on the number of mass points for the optimal input distribution $P_{X^{★}}$ in the scalar case with n=1.

There are several interesting future directions. For example, one interesting direction would be to determine a regime in which a mixture of a mass point at zero and $P_{X_{R}}$ is optimal. It would also be interesting to establish a lower bound on the number of mass points in the support of the optimal input distribution when n=1. We note that such a lower bound was obtained for a point-to-point channel in [30]. We finally remark that the extension of the results of this paper to nondegraded wiretap channels is not trivial and also constitutes an interesting but ambitious future direction.

## Figures and Tables

**Table 1:** Values of ${\bar{R}}_{n}^{\mathrm{MMSE}}\left(1\right)$, ${\bar{R}}_{n}\left(1,\sigma_{2}^{2}\right)$, and ${\bar{R}}_{n}^{\mathrm{ptp}}\left(1\right)$.

*n*	MMSE	$\sigma_{2}^{2}$				ptp
		1.001	1.5	10	1000	
1	1.057	1.057	1.161	1.518	1.664	1.666
2	1.535	1.535	1.687	2.221	2.450	2.454
3	1.908	1.909	2.098	2.768	3.061	3.065
4	2.223	2.224	2.444	3.229	3.575	3.580
5	2.501	2.501	2.750	3.634	4.026	4.031
6	2.751	2.752	3.025	3.999	4.432	4.438
7	2.981	2.982	3.278	4.334	4.805	4.811
8	3.195	3.196	3.513	4.646	5.151	5.158
9	3.395	3.396	3.733	4.937	5.475	5.483
10	3.585	3.586	3.941	5.213	5.781	5.789
11	3.765	3.766	4.139	5.475	6.072	6.080
12	3.936	3.938	4.328	5.725	6.350	6.359
13	4.101	4.102	4.509	5.964	6.616	6.625
14	4.259	4.260	4.683	6.195	6.872	6.881
15	4.412	4.413	4.851	6.417	7.119	7.128
16	4.560	4.561	5.013	6.632	7.357	7.367
17	4.702	4.704	5.170	6.839	7.588	7.598
18	4.841	4.842	5.323	7.041	7.812	7.823
19	4.976	4.977	5.471	7.238	8.030	8.041
20	5.107	5.109	5.616	7.429	8.242	8.254
21	5.235	5.237	5.756	7.615	8.449	8.461
22	5.360	5.362	5.894	7.797	8.651	8.663
23	5.483	5.484	6.028	7.974	8.848	8.860
24	5.602	5.603	6.159	8.148	9.041	9.054
25	5.719	5.720	6.288	8.318	9.230	9.243
26	5.834	5.835	6.414	8.485	9.416	9.428
27	5.946	5.948	6.538	8.649	9.597	9.610
28	6.056	6.058	6.659	8.809	9.775	9.789
29	6.165	6.166	6.778	8.967	9.951	9.964
30	6.271	6.273	6.895	9.122	10.123	10.136
31	6.376	6.378	7.010	9.274	10.292	10.306
32	6.479	6.481	7.124	9.424	10.458	10.472
33	6.580	6.582	7.235	9.571	10.622	10.636
34	6.680	6.682	7.345	9.717	10.783	10.798
35	6.779	6.780	7.453	9.860	10.942	10.957

**Figure A1:**
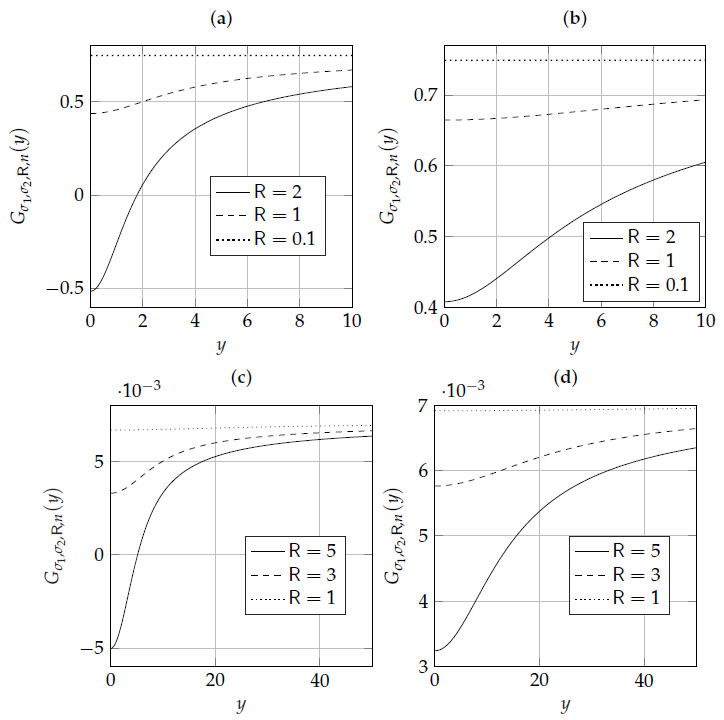
Examples of the function $G_{\sigma_{1},\sigma_{2},R,n}$ defined in (39). (**a**) n=3, $\sigma_{1}=1$, and $\sigma_{2}=2$. (**b**) n=11, $\sigma_{1}=1$, and $\sigma_{2}=2$. (**c**) n=4, $\sigma_{1}=3$, and $\sigma_{2}=3.1$. (**d**) n=11, $\sigma_{1}=3$, and $\sigma_{2}=3.1$.

## Data Availability

Datasets for the numerical results provided in this work are available at [1].

## Appendix A. Examples of the Function *G*_*σ*1,*σ*2,R,*n*_

In this section, we give supporting numerical arguments that the function $G_{\sigma_{1},\sigma_{2},R,n}$ defined in (39) has at most one sign change. Figure A1 demonstrates the behavior of the function $G_{\sigma_{1},\sigma_{2},R,n}$. In addition, the code that generates the function $G_{\sigma_{1},\sigma_{2},R,n}$ for various values of $n,\sigma_{1}$, and $\sigma_{2}$ is provided in [1].

## Appendix B. Derivative of the Secrecy-Density

**Lemma** **A1.**
*The derivative of the secrecy density for the input $P_{X_{R}}$ is*

(A1)
$$\begin{array}{c} {\tilde{\Xi }}^{\prime }(∥x∥;P_{∥X_{R}∥})=∥x∥\;E\left[{\tilde{M}}_{2}\left(\sigma_{1}Q_{n+2}\right)-M_{1}\left(\sigma_{1}Q_{n+2}\right)\right] \end{array}$$

*where $Q_{n+2}^{2}$ is a noncentral chi-square random variable with n+2 degrees of freedom and noncentrality parameter $\frac{{∥x∥}^{2}}{\sigma_{1}^{2}}$ and*

(A2)
$$\begin{array}{cc} M_{i}\left(y\right) & =\frac{1}{\sigma_{i}^{2}}\left(\frac{R}{y}h_{\frac{n}{2}}\left(\frac{R}{\sigma_{i}^{2}}y\right)-1\right),\;i\in \left\{1,2\right\} \end{array}$$

(A3)
$$\begin{array}{cc} {\tilde{M}}_{2}\left(y\right) & =E\left[M_{2}(∥y+W∥)\right], \end{array}$$

*where $W∼N(0_{n+2},\left(\sigma_{2}^{2}-\sigma_{1}^{2}\right)I_{n+2})$.*

**Proof.** We start with the secrecy density expressed in spherical coordinates. A quick way to obtain the information densities in this coordinate system is to note that:

$$\begin{array}{cc} \; & I(X;Y_{i}) \end{array}$$

(A4) $$\begin{array}{cc} \; & =h\left(Y_{i}\right)-h\left(N_{i}\right) \end{array}$$

(A5) $$\begin{array}{cc} \; & =h(∥Y_{i}∥)+\left(n-1\right)E[\log∥Y_{i}∥]+h_{\lambda }\left(\frac{Y_{i}}{∥Y_{i}∥}\right)-h\left(N_{i}\right) \end{array}$$

(A6) $$\begin{array}{cc} \; & =h(∥Y_{i}{∥}^{2})+\left(\frac{n}{2}-1\right)E[\log∥Y_{i}{∥}^{2}]+\log\frac{\pi^{\frac{n}{2}}}{\Gamma \left(\frac{n}{2}\right)}-\frac{n}{2}\log\left(2\pi e\sigma_{i}^{2}\right) \end{array}$$

(A7) $$\begin{array}{cc} \; & =h\left(\sigma_{i}^{2}{∥\frac{X}{\sigma_{i}}+{\tilde{N}}_{i}∥}^{2}\right)+\left(\frac{n}{2}-1\right)E\left[\log\left(\sigma_{i}^{2}{∥\frac{X}{\sigma_{i}}+{\tilde{N}}_{i}∥}^{2}\right)\right]+\log\frac{\pi^{\frac{n}{2}}}{\Gamma \left(\frac{n}{2}\right)}-\frac{n}{2}\log\left(2\pi e\sigma_{i}^{2}\right) \end{array}$$

(A8) $$\begin{array}{cc} \; & =h\left({∥\frac{X}{\sigma_{i}}+{\tilde{N}}_{i}∥}^{2}\right)+\left(\frac{n}{2}-1\right)E\left[\log{∥\frac{X}{\sigma_{i}}+{\tilde{N}}_{i}∥}^{2}\right]-\log\left({\left(2e\right)}^{\frac{n}{2}}\Gamma \left(\frac{n}{2}\right)\right), \end{array}$$

where (A5) holds by [47], Lemma 6.17, and by independence between $∥Y_{i}∥$ and $\frac{Y_{i}}{∥Y_{i}∥}$; the term $h_{\lambda }\left(\cdot \right)$ is a differential entropy-like quantity for random vectors on the *n*-dimensional unit sphere ([47], Lemma 6.16); (A6) holds because $\frac{Y_{i}}{∥Y_{i}∥}$ is uniform on the unit sphere and thanks to [47], Lemma 6.15; the term Γ(z) is the gamma function; and in (A7) we have ${\tilde{N}}_{i}∼N\left(0_{n},I_{n}\right)$. It is now required to write the secrecy density as follows:

(A9) $$\tilde{\Xi }(∥x∥;P_{∥X∥})=i_{1}(∥x∥;P_{X})-i_{2}(∥x∥;P_{X})$$

where

(A10) $$\begin{array}{cc} i_{j}(∥x∥;P_{X}) & =-\int_{0}^{\infty }f_{\chi_{n}^{2}\left(\frac{{∥x∥}^{2}}{\sigma_{j}^{2}}\right)}\left(y\right)\log\frac{\int_{0}^{R}f_{\chi_{n}^{2}\left(\frac{t^{2}}{\sigma_{j}^{2}}\right)}\left(y\right)dP_{∥X∥}\left(t\right)}{y^{\frac{n}{2}-1}}dy-\log\left({\left(2e\right)}^{\frac{n}{2}}\Gamma \left(\frac{n}{2}\right)\right), \end{array}$$

for j∈{1,2}. The term $f_{\chi_{n}^{2}\left(\lambda \right)}\left(y\right)$ is the noncentral chi-square pdf with *n* degrees of freedom and noncentrality parameter λ. Given two values $\rho_{1},\rho_{2}$ with $\rho_{1}>\rho_{2}$, write

(A11) $$\begin{array}{cc} i_{j}\left(\rho_{1};P_{X}\right)-i_{j}\left(\rho_{2};P_{X}\right) & =\int_{0}^{\infty }\left(f_{\chi_{n}^{2}\left(\frac{\rho_{1}^{2}}{\sigma_{j}^{2}}\right)}\left(y\right)-f_{\chi_{n}^{2}\left(\frac{\rho_{2}^{2}}{\sigma_{j}^{2}}\right)}\left(y\right)\right)\log\frac{y^{\frac{n}{2}-1}}{f_{∥\frac{Y}{\sigma_{j}}{∥}^{2}}\left(y;P_{X}\right)}dy \end{array}$$

(A12) $$\begin{array}{cc} \; & =\int_{0}^{\infty }\left(F_{\chi_{n}^{2}\left(\frac{\rho_{2}^{2}}{\sigma_{j}^{2}}\right)}\left(y\right)-F_{\chi_{n}^{2}\left(\frac{\rho_{1}^{2}}{\sigma_{j}^{2}}\right)}\left(y\right)\right)\frac{d}{dy}\log\frac{y^{\frac{n}{2}-1}}{f_{∥\frac{Y}{\sigma_{j}}{∥}^{2}}\left(y;P_{X}\right)}dy \end{array}$$

where we have integrated by parts and where $F_{\chi_{n}^{2}\left(\lambda \right)}\left(y\right)$ is the cumulative distribution function of $\chi_{n}^{2}\left(\lambda \right)$. Now notice that

(A13) $$\int_{0}^{\infty }\left(F_{\chi_{n}^{2}\left(\frac{\rho_{2}^{2}}{\sigma_{j}^{2}}\right)}\left(y\right)-F_{\chi_{n}^{2}\left(\frac{\rho_{1}^{2}}{\sigma_{j}^{2}}\right)}\left(y\right)\right)dy=\frac{\rho_{1}^{2}-\rho_{2}^{2}}{\sigma_{j}^{2}}.$$

Since $\chi_{n}^{2}\left(\frac{\rho_{1}^{2}}{\sigma_{j}^{2}}\right)$ statistically dominates $\chi_{n}^{2}\left(\frac{\rho_{2}^{2}}{\sigma_{j}^{2}}\right)$, the integrand function in (A13) is always positive. We can introduce an auxiliary output random variable $Q_{j}$, for j∈{1,2}, with pdf

(A14) $$f_{Q_{j}}\left(y;\rho_{1},\rho_{2}\right)=\frac{\sigma_{j}^{2}}{\rho_{1}^{2}-\rho_{2}^{2}}\left(F_{\chi_{n}^{2}\left(\frac{\rho_{2}^{2}}{\sigma_{j}^{2}}\right)}\left(y\right)-F_{\chi_{n}^{2}\left(\frac{\rho_{1}^{2}}{\sigma_{j}^{2}}\right)}\left(y\right)\right),$$

for y>0, to rewrite (A12) as follows:

(A15) $$\begin{array}{cc} i_{j}\left(\rho_{1};P_{X}\right)-i_{j}\left(\rho_{2};P_{X}\right) & =-\frac{\rho_{1}^{2}-\rho_{2}^{2}}{\sigma_{j}^{2}}\int_{0}^{\infty }f_{Q_{j}}\left(y;\rho_{1},\rho_{2}\right)\frac{d}{dy}\log\frac{f_{∥\frac{Y}{\sigma_{j}}{∥}^{2}}\left(y;P_{X}\right)}{y^{\frac{n}{2}-1}}dy. \end{array}$$

We evaluate the derivative in (A15) as:

$$\begin{array}{cc} \; & \frac{d}{dy}\log\frac{f_{∥\frac{Y}{\sigma_{j}}{∥}^{2}}\left(y;P_{X}\right)}{y^{\frac{n}{2}-1}} \end{array}$$

(A16) $$\begin{array}{cc} \; & \;=\frac{y^{\frac{n}{2}-1}}{f_{∥\frac{Y}{\sigma_{j}}{∥}^{2}}\left(y;P_{X}\right)}\int_{0}^{R}\frac{d}{dy}\frac{f_{\chi_{n}^{2}\left(\frac{t^{2}}{\sigma_{j}^{2}}\right)}\left(y\right)}{y^{\frac{n}{2}-1}}dP_{∥X∥}\left(t\right) \end{array}$$

(A17) $$\begin{array}{cc} \; & \;=\frac{y^{\frac{n}{2}-1}}{f_{∥\frac{Y}{\sigma_{j}}{∥}^{2}}\left(y;P_{X}\right)}\int_{0}^{R}\left(\frac{f_{\chi_{n-2}^{2}\left(\frac{t^{2}}{\sigma_{j}^{2}}\right)}\left(y\right)}{2y^{\frac{n}{2}-1}}-\left(\frac{1}{2}+\frac{\frac{n}{2}-1}{y}\right)\frac{f_{\chi_{n}^{2}\left(\frac{t^{2}}{\sigma_{j}^{2}}\right)}\left(y\right)}{y^{\frac{n}{2}-1}}\right)dP_{∥X∥}\left(t\right) \end{array}$$

(A18) $$\begin{array}{cc} \; & \;=E\left[\frac{1}{2}\frac{f_{\chi_{n-2}^{2}\left(\frac{{∥X∥}^{2}}{\sigma_{j}^{2}}\right)}\left(\frac{{∥Y∥}^{2}}{\sigma_{j}^{2}}\right)}{f_{\chi_{n}^{2}\left(\frac{{∥X∥}^{2}}{\sigma_{j}^{2}}\right)}\left(\frac{{∥Y∥}^{2}}{\sigma_{j}^{2}}\right)}-\left(\frac{1}{2}+\frac{\frac{n}{2}-1}{\frac{{∥Y∥}^{2}}{\sigma_{j}^{2}}}\right)|\frac{{∥Y∥}^{2}}{\sigma_{j}^{2}}=y\right] \end{array}$$

(A19) $$\begin{array}{cc} \; & \;=E\left[\frac{1}{2}\frac{∥X∥}{∥Y∥}\frac{I_{\frac{n}{2}-2}\left(\frac{∥X∥∥Y∥}{\sigma_{j}^{2}}\right)}{I_{\frac{n}{2}-1}\left(\frac{∥X∥∥Y∥}{\sigma_{j}^{2}}\right)}-\left(\frac{1}{2}+\frac{\frac{n}{2}-1}{\frac{{∥Y∥}^{2}}{\sigma_{j}^{2}}}\right)|\frac{{∥Y∥}^{2}}{\sigma_{j}^{2}}=y\right] \end{array}$$

(A20) $$\begin{array}{cc} \; & \;=E\left[\frac{1}{2}\frac{∥X∥}{∥Y∥}h_{\frac{n}{2}}\left(\frac{∥X∥∥Y∥}{\sigma_{j}^{2}}\right)-\frac{1}{2}|\frac{{∥Y∥}^{2}}{\sigma_{j}^{2}}=y\right] \end{array}$$

where, in (A16), we used

(A21) $$f_{∥\frac{Y}{\sigma_{j}}{∥}^{2}}\left(y;P_{X}\right)=\int_{0}^{R}f_{\chi_{n}^{2}\left(\frac{t^{2}}{\sigma_{j}^{2}}\right)}\left(y\right)dP_{∥X∥}\left(t\right);$$

in (A17), we used the relationship

(A22) $$\frac{d}{dy}f_{\chi_{n}^{2}\left(\rho^{2}\right)}\left(y\right)=\frac{1}{2}f_{\chi_{n-2}^{2}\left(\rho^{2}\right)}\left(y\right)-\frac{1}{2}f_{\chi_{n}^{2}\left(\rho^{2}\right)}\left(y\right);$$

and (A20) follows from the recurrence relationship

(A23) $$I_{\nu -1}\left(z\right)-I_{\nu +1}\left(z\right)=\frac{2\nu }{z}I_{\nu }\left(z\right).$$

Putting together (A15) and (A20), we find

(A24) $$\begin{array}{cc} i_{j}\left(\rho_{1};P_{X}\right)-i_{j}\left(\rho_{2};P_{X}\right) & =-\frac{\rho_{1}^{2}-\rho_{2}^{2}}{2\sigma_{j}^{2}}E\left[E\left[\frac{∥X∥}{∥Y∥}h_{\frac{n}{2}}\left(\frac{∥X∥∥Y∥}{\sigma_{j}^{2}}\right)-1|\frac{{∥Y∥}^{2}}{\sigma_{j}^{2}}=Q_{j}\right]\right]. \end{array}$$

We are now in the position to compute the derivative of the information density as

(A25) $$\begin{array}{cc} i_{j}^{\prime }\left(\rho ;P_{X}\right) & =\lim_{h\to 0}\frac{i_{j}\left(\rho +h;P_{X}\right)-i_{j}\left(\rho ;P_{X}\right)}{h} \end{array}$$

(A26) $$\begin{array}{cc} \; & =-\frac{\rho }{\sigma_{j}^{2}}\;E\left[E\left[\frac{∥X∥}{∥Y∥}h_{\frac{n}{2}}\left(\frac{∥X∥∥Y∥}{\sigma_{j}^{2}}\right)-1|\frac{{∥Y∥}^{2}}{\sigma_{j}^{2}}=Q^{\prime }\right]\right], \end{array}$$

where $Q^{\prime }∼\chi_{n+2}^{2}\left(\frac{\rho^{2}}{\sigma_{j}^{2}}\right)$ thanks to Lemma A2. The final result is obtained by letting

(A27) $$\begin{array}{c} {\tilde{\Xi }}^{\prime }(∥x∥;P_{∥x∥})=i_{1}^{\prime }(∥x∥;P_{X})-i_{2}^{\prime }(∥x∥;P_{X}) \end{array}$$

and by specializing the result to the input $P_{X_{R}}$. □

**Lemma** **A2.**
*Consider the pdf $f_{Q_{j}}\left(y;\rho_{1},\rho_{2}\right)$ defined in (A14). For any ρ≥0 we have*

(A28)
$$\lim_{h\to 0}f_{Q_{j}}\left(y;\rho +h,\rho \right)=f_{\chi_{n+2}^{2}\left(\frac{\rho^{2}}{\sigma_{j}^{2}}\right)}\left(y\right),\;y>0.$$

**Proof.** Thanks to the definition (A14), we have

$$\begin{array}{cc} \; & \lim_{h\to 0}f_{Q_{j}}\left(y;\rho +h,\rho \right) \end{array}$$

(A29) $$\begin{array}{cc} \; & \;=\lim_{h\to 0}\frac{\sigma_{j}^{2}}{h(2\rho +h)}\left(F_{\chi_{n}^{2}\left(\frac{\rho^{2}}{\sigma_{j}^{2}}\right)}\left(y\right)-F_{\chi_{n}^{2}\left(\frac{{\left(\rho +h\right)}^{2}}{\sigma_{j}^{2}}\right)}\left(y\right)\right) \end{array}$$

(A30) $$\begin{array}{cc} \; & \;=\lim_{h\to 0}\frac{\sigma_{j}^{2}}{h(2\rho +h)}\int_{0}^{y}\left(f_{\chi_{n}^{2}\left(\frac{\rho^{2}}{\sigma_{j}^{2}}\right)}\left(t\right)-f_{\chi_{n}^{2}\left(\frac{{\left(\rho +h\right)}^{2}}{\sigma_{j}^{2}}\right)}\left(t\right)\right)dt \end{array}$$

(A31) $$\begin{array}{cc} \; & \;=\frac{\sigma_{j}^{2}}{2\rho }\int_{0}^{y}\sum_{i=0}^{\infty }\lim_{h\to 0}\frac{1}{h}\left(\frac{e^{-\frac{\rho^{2}}{2\sigma_{j}^{2}}}{\left(\frac{\rho^{2}}{2\sigma_{j}^{2}}\right)}^{i}}{i!}-\frac{e^{-\frac{{\left(\rho +h\right)}^{2}}{2\sigma_{j}^{2}}}{\left(\frac{{\left(\rho +h\right)}^{2}}{2\sigma_{j}^{2}}\right)}^{i}}{i!}\right)f_{\chi_{n+2i}^{2}}\left(t\right)dt \end{array}$$

(A32) $$\begin{array}{cc} \; & \;=\frac{\sigma_{j}^{2}}{2\rho }\int_{0}^{y}\sum_{i=0}^{\infty }\frac{d}{d\rho }\left(\frac{e^{-\frac{\rho^{2}}{2\sigma_{j}^{2}}}{\left(\frac{\rho^{2}}{2\sigma_{j}^{2}}\right)}^{i}}{i!}\right)f_{\chi_{n+2i}^{2}}\left(t\right)dt \end{array}$$

(A33) $$\begin{array}{cc} \; & \;=\frac{1}{2}\int_{0}^{y}\sum_{i=0}^{\infty }\left(-\frac{e^{-\frac{\rho^{2}}{2\sigma_{j}^{2}}}{\left(\frac{\rho^{2}}{2\sigma_{j}^{2}}\right)}^{i}}{i!}+\frac{e^{-\frac{\rho^{2}}{2\sigma_{j}^{2}}}{\left(\frac{\rho^{2}}{2\sigma_{j}^{2}}\right)}^{i-1}}{(i-1)!}1\left(i\geq 1\right)\right)f_{\chi_{n+2i}^{2}}\left(t\right)dt \end{array}$$

(A34) $$\begin{array}{cc} \; & \;=\frac{1}{2}\int_{0}^{y}\left(-f_{\chi_{n}^{2}\left(\frac{\rho^{2}}{\sigma_{j}^{2}}\right)}\left(t\right)+f_{\chi_{n+2}^{2}\left(\frac{\rho^{2}}{\sigma_{j}^{2}}\right)}\left(t\right)\right)dt \end{array}$$

(A35) $$\begin{array}{cc} \; & \;=\int_{0}^{y}\frac{d}{dt}f_{\chi_{n+2}^{2}\left(\frac{\rho^{2}}{\sigma_{j}^{2}}\right)}\left(t\right)dt \end{array}$$

(A36) $$\begin{array}{cc} \; & \;=f_{\chi_{n+2}^{2}\left(\frac{\rho^{2}}{\sigma_{j}^{2}}\right)}\left(y\right), \end{array}$$

where 1(·) is the indicator function; in (A31) we used the Poisson-weighted mixture representation of the noncentral chi-square pdf, and in (A35), we used (A22). □

**Lemma** **A3.**
*There exists some $L=L(\sigma_{1},\sigma_{2},R)<\infty$ such that*

(A37)
$$\begin{array}{cc} \; & N\left(R,g\left(\cdot \right)+\log\left(\frac{\sigma_{2}}{\sigma_{1}}\right)-C_{s}\right)=N\left(\left[-L,L\right],g\left(\cdot \right)+\log\left(\frac{\sigma_{2}}{\sigma_{1}}\right)-C_{s}\right)<\infty . \end{array}$$

*Furthermore, L can be upper-bounded as follows:*

(A38)
$$L\leq Rd_{1}+d_{2}$$

*where*

(A39)
$$\begin{array}{cc} d_{1} & =\frac{\sigma_{2}+\sigma_{1}}{\sigma_{2}-\sigma_{1}}, \end{array}$$

(A40)
$$\begin{array}{cc} d_{2} & =\sqrt{\frac{\frac{\sigma_{2}^{2}-\sigma_{1}^{2}}{\sigma_{2}^{2}}+2C_{s}}{\frac{1}{\sigma_{1}^{2}}-\frac{1}{\sigma_{2}^{2}}}}\leq \sqrt{\frac{\frac{\sigma_{2}^{2}-\sigma_{1}^{2}}{\sigma_{2}^{2}}+2C_{G}}{\frac{1}{\sigma_{1}^{2}}-\frac{1}{\sigma_{2}^{2}}}}, \end{array}$$

*with*

(A41)
$$\begin{array}{cc} C_{G}\left(\sigma_{1}^{2},\sigma_{2}^{2},R^{2},1\right) & =\frac{1}{2}\log\frac{1+R^{2}/\sigma_{1}^{2}}{1+R^{2}/\sigma_{2}^{2}}. \end{array}$$

**Proof.** First, note that $C_{s}\leq C_{G}$ thanks to (69). Second, for |y|≥R, we can lower-bound the function *g* as follows:

(A42) $$\begin{array}{cc} g(y) & =E\left[\log f_{Y_{2}^{★}}\left(y+N\right)\right]-\log f_{Y_{1}^{★}}\left(y\right) \end{array}$$

(A43) $$\begin{array}{cc} \; & =E\left[\log E[\phi_{\sigma_{2}}\left(y+N-X^{★}\right)|N]\right]-\log E\left[\phi_{\sigma_{1}}\left(y-X^{★}\right)\right] \end{array}$$

(A44) $$\begin{array}{cc} \; & \geq E\left[\log\phi_{\sigma_{2}}\left(y+N-X^{★}\right)\right]-\log E\left[\phi_{\sigma_{1}}\left(y-X^{★}\right)\right] \end{array}$$

(A45) $$\begin{array}{cc} \; & \geq \log\frac{\sigma_{1}}{\sigma_{2}}-E\left[\frac{{\left(y+N-X^{★}\right)}^{2}}{2\sigma_{2}^{2}}\right]+\frac{{\left(|y|-R\right)}^{2}}{2\sigma_{1}^{2}} \end{array}$$

(A46) $$\begin{array}{cc} \; & =\log\frac{\sigma_{1}}{\sigma_{2}}-E\left[\frac{{\left(y-X^{★}\right)}^{2}}{2\sigma_{2}^{2}}\right]-\frac{\sigma_{2}^{2}-\sigma_{1}^{2}}{2\sigma_{2}^{2}}+\frac{{\left(|y|-R\right)}^{2}}{2\sigma_{1}^{2}} \end{array}$$

(A47) $$\begin{array}{cc} \; & \geq \log\frac{\sigma_{1}}{\sigma_{2}}-\frac{{\left(|y|+R\right)}^{2}}{2\sigma_{2}^{2}}-\frac{\sigma_{2}^{2}-\sigma_{1}^{2}}{2\sigma_{2}^{2}}+\frac{{\left(|y|-R\right)}^{2}}{2\sigma_{1}^{2}}, \end{array}$$

where (A44) follows from applying Jensen’s inequality and the law of iterated expectation to the first term; (A45) follows from

(A48) $$E\left[\phi_{\sigma_{1}}\left(y-X^{★}\right)\right]\leq \phi_{\sigma_{1}}\left(|y\right|-R),\;|y|\geq R;$$

and (A47) follows from ${\left(y-X^{★}\right)}^{2}\leq {\left(|y|+R\right)}^{2}$ for all $\left|y|\geq R\geq \right|X^{★}|$. The RHS of

(A49) $$\begin{array}{cc} g\left(y\right)+\log\left(\frac{\sigma_{2}}{\sigma_{1}}\right)-C_{s} & \geq -\frac{{\left(|y|+R\right)}^{2}}{2\sigma_{2}^{2}}-\frac{\sigma_{2}^{2}-\sigma_{1}^{2}}{2\sigma_{2}^{2}}+\frac{{\left(|y|-R\right)}^{2}}{2\sigma_{1}^{2}}-C_{s} \end{array}$$

is strictly positive when

(A50) $$|y|>\frac{R\left(\frac{1}{\sigma_{1}^{2}}+\frac{1}{\sigma_{2}^{2}}\right)+\sqrt{\frac{4R^{2}}{\sigma_{1}^{2}\sigma_{2}^{2}}+\left(\frac{1}{\sigma_{1}^{2}}-\frac{1}{\sigma_{2}^{2}}\right)\left(\frac{\sigma_{2}^{2}-\sigma_{1}^{2}}{\sigma_{2}^{2}}+2C_{s}\right)}}{\frac{1}{\sigma_{1}^{2}}-\frac{1}{\sigma_{2}^{2}}}.$$

By using the bound $\sqrt{a+b}\leq \sqrt{a}+\sqrt{b}$, we arrive at

(A51) $$\begin{array}{cc} \left|y\right| & \geq R\frac{\sigma_{2}+\sigma_{1}}{\sigma_{2}-\sigma_{1}}+\sqrt{\frac{\frac{\sigma_{2}^{2}-\sigma_{1}^{2}}{\sigma_{2}^{2}}+2C_{s}}{\frac{1}{\sigma_{1}^{2}}-\frac{1}{\sigma_{2}^{2}}}}. \end{array}$$

This concludes the proof for the bound on *L*. □

**Lemma** **A4.**
*Let $\overset{˘}{h}:C\to C$ denote the complex extension of the function h in (123). Then, for B≥R, we have that*

(A52)
$$\begin{array}{cc} \; & \max_{|z|\leq B}\left|\overset{˘}{h}\left(z\right)\right|\leq \frac{1}{\sqrt{2\pi \sigma_{1}^{2}}}e^{\frac{B^{2}}{2\sigma_{1}^{2}}}\left(a_{1}B^{2}+a_{2}B+a_{3}\right) \end{array}$$

*where*

(A53)
$$\begin{array}{cc} a_{1} & =\frac{3\sigma_{1}^{2}}{\sigma_{2}^{2}\sqrt{\sigma_{2}^{2}-\sigma_{1}^{2}}}, \end{array}$$

(A54)
$$\begin{array}{cc} a_{2} & =\frac{\sqrt{2}\sigma_{1}^{2}}{\sqrt{\sigma_{2}^{2}}\sqrt{\sigma_{2}^{2}-\sigma_{1}^{2}}}+2, \end{array}$$

(A55)
$$\begin{array}{cc} a_{3} & =\frac{\sigma_{1}^{2}}{\sqrt{\sigma_{2}^{2}-\sigma_{1}^{2}}}\left(\sqrt{|\log\left(2\pi \sigma_{2}^{2}\right){|}^{2}+\frac{24{\left(\sigma_{2}^{2}-\sigma_{1}^{2}\right)}^{2}}{\sigma_{2}^{4}}+\pi^{2}}\right). \end{array}$$

**Proof.** Let us denote $z=z_{R}+iz_{I}$, where $z_{R}$ and $z_{I}$ are real numbers and $i=\sqrt{-1}$ is the imaginary unit. Then, by triangular inequality, we have:

(A56) $$\begin{array}{cc} |\overset{˘}{h}\left(z\right)| & =\left|\frac{\sigma_{1}^{2}f_{Y_{1}}\left(z\right)E\left[N\log f_{Y_{2}}\left(z+N\right)\right]}{\sigma_{2}^{2}-\sigma_{1}^{2}}-E\left[X^{★}\phi_{\sigma_{1}}\left(z-X^{★}\right)\right]+zf_{Y_{1}}\left(z\right)\right| \end{array}$$

(A57) $$\begin{array}{cc} \; & \leq \left|f_{Y_{1}}\left(z\right)\right|\left(\frac{\sigma_{1}^{2}}{\sigma_{2}^{2}-\sigma_{1}^{2}}E\left[|N|\cdot |\log f_{Y_{2}}\left(z+N\right)|\right]+\left|z\right|\right)+E\left[|X^{★}\left|\cdot \right|\phi_{\sigma_{1}}\left(z-X^{★}\right)|\right]. \end{array}$$

Next, let us upper-bound each contribution of (A57). For |z|≤B, we have

$$\begin{array}{cc} \; & {\left|\log f_{Y_{2}}\left(z+n\right)\right|}^{2} \end{array}$$

(A58) $$\begin{array}{cc} \; & \;={\left|\log\left|f_{Y_{2}}\left(z+n\right)\right|+i\arg\left(f_{Y_{2}}\left(z+n\right)\right)\right|}^{2} \end{array}$$

(A59) $$\begin{array}{cc} \; & \;=\log^{2}\left|f_{Y_{2}}\left(z+n\right)\right|+\arg^{2}\left(f_{Y_{2}}\left(z+n\right)\right) \end{array}$$

(A60) $$\begin{array}{cc} \; & \;=\log^{2}\left|E\left[\phi_{\sigma_{2}}\left(z+n-X^{★}\right)\right]\right|+\arg^{2}\left(E\left[\phi_{\sigma_{2}}\left(z+n-X^{★}\right)\right]\right) \end{array}$$

(A61) $$\begin{array}{cc} \; & \;\leq \log^{2}\left(\frac{1}{\sqrt{2\pi \sigma_{2}^{2}}}E\left[\exp\left(-\frac{{\left(z_{R}+n-X^{★}\right)}^{2}-z_{I}^{2}}{2\sigma_{2}^{2}}\right)\right]\right)+\arg^{2}\left(\sum_{x}\alpha_{x}\exp\left(i\theta_{x}\right)\right) \end{array}$$

(A62) $$\begin{array}{cc} \; & \;\leq {\left(\frac{z_{I}^{2}}{2\sigma_{2}^{2}}-\frac{1}{2}\log\left(2\pi \sigma_{2}^{2}\right)+\log E\left[e^{-\frac{{\left(z_{R}+n-X^{★}\right)}^{2}}{2\sigma_{2}^{2}}}\right]\right)}^{2}+\pi^{2} \end{array}$$

(A63) $$\begin{array}{cc} \; & \;\leq 2\;{\left(\;\frac{z_{I}^{2}}{2\sigma_{2}^{2}}-\frac{1}{2}\log\left(2\pi \sigma_{2}^{2}\right)\right)}^{2}+\;2\log^{2}E\left[e^{-\frac{{\left(z_{R}+n-X^{★}\right)}^{2}}{2\sigma_{2}^{2}}}\right]\;+\;\pi^{2} \end{array}$$

(A64) $$\begin{array}{cc} \; & \;\leq 2{\left(\frac{z_{I}^{2}}{2\sigma_{2}^{2}}-\frac{1}{2}\log\left(2\pi \sigma_{2}^{2}\right)\right)}^{2}+2\;\frac{E^{2}\left[{\left(z_{R}+n-X^{★}\right)}^{2}\right]}{4\sigma_{2}^{4}}+\pi^{2} \end{array}$$

(A65) $$\begin{array}{cc} \; & \;\leq 2{\left(\frac{z_{I}^{2}}{2\sigma_{2}^{2}}-\frac{1}{2}\log\left(2\pi \sigma_{2}^{2}\right)\right)}^{2}+2\frac{{\left({\left(z_{R}+n\right)}^{2}+R^{2}\right)}^{2}}{4\sigma_{2}^{4}}+\pi^{2} \end{array}$$

(A66) $$\begin{array}{cc} \; & \;\leq \frac{2B^{2}}{\sigma_{2}^{2}}+{\left|\log\left(2\pi \sigma_{2}^{2}\right)\right|}^{2}+\frac{8\left(B^{4}+n^{4}\right)+R^{4}}{\sigma_{2}^{4}}+\pi^{2}, \end{array}$$

where step (A61) holds by triangular inequality; step (A62) holds by noticing that

(A67) $$-\pi <\arg\left(\sum_{x\in \mathrm{supp}(P_{X^{★}})}\alpha_{x}\exp\left(i\theta_{x}\right)\right)\leq \pi ,$$

where $\left\{\alpha_{x}\right\}$ and $\left\{\theta_{x}\right\}$ are real numbers that depend on *x*; (A63) follows from using the bound ${\left(a+b\right)}^{2}\leq 2\left(a^{2}+b^{2}\right)$; (A64) holds because $x\mapsto \log^{2}\left(x\right)$ is a decreasing function for x<1 and because $E\left[e^{-\frac{{\left(z_{R}+n-X^{★}\right)}^{2}}{2\sigma_{2}^{2}}}\right]\geq e^{-\frac{E\left[{\left(z_{R}+n-X^{★}\right)}^{2}\right]}{2\sigma_{2}^{2}}}$, which follows from Jensen’s inequality; (A65) follows from $E[X^{★}]=0$ and $E\left[{\left(X^{★}\right)}^{2}\right]\leq R^{2}$; and (A66) follows from the bound ${\left|a+b\right|}^{k}\leq 2^{k-1}{\left(|a\right|}^{k}+{\left|b\right|}^{k})$ for k≥1. Furthermore, given that $|z_{R}|\leq B$ and $|z_{I}|\leq B$, we arrive at the bound

(A68) $${\left({\left(z_{R}+n\right)}^{2}+R^{2}\right)}^{2}\leq 2\left(8\left(B^{4}+n^{4}\right)+R^{4}\right).$$

Consequently,

$$\begin{array}{cc} \; & \frac{E\left[|N|\cdot |\log f_{Y_{2}}\left(z+N\right)|\right]}{\sqrt{\sigma_{2}^{2}-\sigma_{1}^{2}}} \end{array}$$

(A69) $$\begin{array}{cc} \; & \;\leq \frac{\sqrt{E\left[{\left|N\right|}^{2}\right]E\left[|\log f_{Y_{2}}{\left(z+N\right)|}^{2}\right]}}{\sqrt{\sigma_{2}^{2}-\sigma_{1}^{2}}} \end{array}$$

(A70) $$\begin{array}{cc} \; & \;\leq \sqrt{\frac{2B^{2}}{\sigma_{2}^{2}}+{\left|\log\left(2\pi \sigma_{2}^{2}\right)\right|}^{2}+\frac{8\left(B^{4}+E\left[N^{4}\right]\right)+R^{4}}{\sigma_{2}^{4}}+\pi^{2}} \end{array}$$

(A71) $$\begin{array}{cc} \; & \;=\sqrt{\frac{2B^{2}}{\sigma_{2}^{2}}+{\left|\log\left(2\pi \sigma_{2}^{2}\right)\right|}^{2}+\frac{8B^{4}+24{\left(\sigma_{2}^{2}-\sigma_{1}^{2}\right)}^{2}+R^{4}}{\sigma_{2}^{4}}+\pi^{2}}, \end{array}$$

where (A69) follows from Cauchy–Schwarz inequality; (A70) follows from $E\left[N^{4}\right]=3{\left(\sigma_{2}^{2}-\sigma_{1}^{2}\right)}^{2}$. Moreover, we have

(A72) $$\begin{array}{cc} |f_{Y_{1}}\left(z\right)| & \leq E\left[\left|\phi_{\sigma_{1}}\left(z-X^{★}\right)\right|\right] \end{array}$$

(A73) $$\begin{array}{cc} \; & =\frac{1}{\sqrt{2\pi \sigma_{1}^{2}}}E\left[\exp\left(-\frac{{\left(z_{R}-X^{★}\right)}^{2}-z_{I}^{2}}{2\sigma_{1}^{2}}\right)\right] \end{array}$$

(A74) $$\begin{array}{cc} \; & \leq \frac{1}{\sqrt{2\pi \sigma_{1}^{2}}}\exp\left(\frac{B^{2}}{2\sigma_{1}^{2}}\right), \end{array}$$

and finally

(A75) $$\begin{array}{cc} E\left[|X^{★}\left|\cdot \right|\phi_{\sigma_{1}}\left(z-X^{★}\right)|\right] & \leq R\;E\left[|\phi_{\sigma_{1}}\left(z-X^{★}\right)|\right] \end{array}$$

(A76) $$\begin{array}{cc} \; & \leq R\frac{1}{\sqrt{2\pi \sigma_{1}^{2}}}\exp\left(\frac{B^{2}}{2\sigma_{1}^{2}}\right). \end{array}$$

Putting all contributions together, we get

(A77) $$\begin{array}{cc} |\overset{˘}{h}\left(z\right)|\sqrt{2\pi \sigma_{1}^{2}}e^{-\frac{B^{2}}{2\sigma_{1}^{2}}} & \leq \frac{\sigma_{1}^{2}\sqrt{\frac{2B^{2}}{\sigma_{2}^{2}}+{\left|\log\left(2\pi \sigma_{2}^{2}\right)\right|}^{2}+\frac{8B^{4}+24{\left(\sigma_{2}^{2}-\sigma_{1}^{2}\right)}^{2}+R^{4}}{\sigma_{2}^{4}}+\pi^{2}}}{\sqrt{\sigma_{2}^{2}-\sigma_{1}^{2}}}+B+R \end{array}$$

(A78) $$\begin{array}{cc} \; & \leq a_{1}B^{2}+a_{2}B+a_{3}, \end{array}$$

where, in the last step, we have used that $\sqrt{\sum_{i}x_{i}}\leq \sum_{i}\sqrt{x_{i}}$ and the fact that R≤B. □

**Lemma** **A5.** *Let $\overset{˘}{h}:C\to C$ denote the complex extension of the function h in (123). Then, for*

(A79) $$\begin{array}{c} B\geq R\frac{\sigma_{2}^{2}+\sigma_{1}^{2}}{\sigma_{2}^{2}-\sigma_{1}^{2}}, \end{array}$$

*we have that*

(A80) $$\begin{array}{c} \max_{|z|\leq B}\left|\overset{˘}{h}\left(z\right)\right|\geq \left(c_{1}B-c_{2}R\right)\frac{\exp\left(-\frac{{\left(B+R\right)}^{2}}{2\sigma_{1}^{2}}\;\right)}{\sqrt{2\pi \sigma_{1}^{2}}}>0, \end{array}$$

*where $c_{1}=1-\frac{\sigma_{1}^{2}}{\sigma_{2}^{2}}$ and* $c_{2}=1+\frac{\sigma_{1}^{2}}{\sigma_{2}^{2}}$.

**Proof.** First, note that

(A81) $$\begin{array}{c} \frac{E_{N}\left[E[X^{★}|Y_{2}=B+N]\right]}{\sigma_{2}^{2}}-\frac{E[X^{★}|Y_{1}=B]}{\sigma_{1}^{2}}\geq -\frac{R}{\sigma_{2}^{2}}-\frac{R}{\sigma_{1}^{2}}. \end{array}$$

Second, note that the condition in (A79) implies that

(A82) $$\begin{array}{c} 0\leq B\left(\frac{1}{\sigma_{1}^{2}}-\frac{1}{\sigma_{2}^{2}}\right)-\frac{R}{\sigma_{2}^{2}}-\frac{R}{\sigma_{1}^{2}}. \end{array}$$

Therefore, by using (111) together with (A81) and (A82), we arrive at

(A83) $$\begin{array}{cc} \max_{|z|\leq B}\left|\overset{˘}{h}\left(z\right)\right| & \geq \left|\overset{˘}{h}\left(B\right)\right| \end{array}$$

(A84) $$\begin{array}{cc} \; & =\left|\frac{E\left[E[X^{★}|Y_{2}=B+N]\right]-B}{\sigma_{2}^{2}}-\frac{E[X^{★}|Y_{1}=B]-B}{\sigma_{1}^{2}}\right|\sigma_{1}^{2}f_{Y_{1}}\left(B\right) \end{array}$$

(A85) $$\begin{array}{cc} \; & \geq \left(B\left(\frac{1}{\sigma_{1}^{2}}-\frac{1}{\sigma_{2}^{2}}\right)-\frac{R}{\sigma_{2}^{2}}-\frac{R}{\sigma_{1}^{2}}\right)\sigma_{1}^{2}f_{Y_{1}}\left(B\right) \end{array}$$

(A86) $$\begin{array}{cc} \; & \geq \left(B\left(\frac{1}{\sigma_{1}^{2}}-\frac{1}{\sigma_{2}^{2}}\right)-\frac{R}{\sigma_{2}^{2}}-\frac{R}{\sigma_{1}^{2}}\right)\frac{\sigma_{1}^{2}}{\sqrt{2\pi \sigma_{1}^{2}}}\exp\left(-\frac{{\left(B+R\right)}^{2}}{2\sigma_{1}^{2}}\right), \end{array}$$

where in last bound we have used Jensen’s inequality to arrive at

(A87) $$\begin{array}{cc} f_{Y_{1}}\left(B\right) & =E\left[\phi_{\sigma_{1}}\left(B-X^{★}\right)\right] \end{array}$$

(A88) $$\begin{array}{cc} \; & =\frac{1}{\sqrt{2\pi \sigma_{1}^{2}}}E\left[\exp\left(-\frac{{\left(B-X^{★}\right)}^{2}}{2\sigma_{1}^{2}}\right)\right] \end{array}$$

(A89) $$\begin{array}{cc} \; & \geq \frac{1}{\sqrt{2\pi \sigma_{1}^{2}}}\exp\left(-\frac{{\left(B+R\right)}^{2}}{2\sigma_{1}^{2}}\right). \end{array}$$

This concludes the proof. □

## Appendix C. Proof of Theorem 7

To study the large *n* behavior, we need the following bounds on the function $h_{\nu }$ [39,40]: for $\nu >\frac{1}{2}$

(A90) $$\begin{array}{c} h_{\nu }\left(x\right)=\frac{x}{\frac{2\nu -1}{2}+\sqrt{\frac{{\left(2\nu -1\right)}^{2}}{4}+x^{2}}}\cdot g_{\nu }\left(x\right), \end{array}$$

where

(A91) $$\begin{array}{c} 1\geq g_{\nu }\left(x\right)\geq \frac{\frac{2\nu -1}{2}+\sqrt{\frac{{\left(2\nu -1\right)}^{2}}{4}+x^{2}}}{\nu +\sqrt{\nu^{2}+x^{2}}}. \end{array}$$

Moreover, let

(A92) $$U_{n}=∥R+\sqrt{s}Z∥$$

with $Z∼N(0_{n},\sigma^{2}I_{n})$. Consequently,

$$\begin{array}{cc} \; & \lim_{n\to \infty }E\left[h_{\frac{n}{2}}^{2}\left(\frac{∥R+\sqrt{s}Z∥R}{s}\right)\right] \end{array}$$

(A93) $$\begin{array}{cc} \; & \;=E\left[\lim_{n\to \infty }h_{\frac{n}{2}}^{2}\left(\frac{∥R+\sqrt{s}Z∥R}{s}\right)\right] \end{array}$$

(A94) $$\begin{array}{cc} \; & \;=E\left[\lim_{n\to \infty }\frac{U_{n}^{2}\frac{R^{2}}{s^{2}}}{{\left(\frac{n-1}{2}+\sqrt{\frac{{\left(n-1\right)}^{2}}{4}+U_{n}^{2}\frac{R^{2}}{s^{2}}}\right)}^{2}}\cdot g_{\frac{n}{2}}^{2}\left(U_{n}\frac{R}{s}\right)\right] \end{array}$$

(A95) $$\begin{array}{cc} \; & \;=E\left[\lim_{n\to \infty }\frac{\frac{1}{n}U_{n}^{2}\frac{R^{2}}{s^{2}}}{n\cdot {\left(\frac{1}{2}+\sqrt{\frac{1}{4}+{\left(\frac{1}{n}U_{n}\frac{R}{s}\right)}^{2}}\right)}^{2}}\cdot g_{\frac{n}{2}}^{2}\left(U_{n}\frac{R}{s}\right)\right] \end{array}$$

(A96) $$\begin{array}{cc} \; & \;=0, \end{array}$$

where (A93) follows from the dominated convergence theorem, since $|h_{\nu }|\leq 1$; (A94) follows from using (A90); (A96) follows from using the strong law of large numbers to note that

(A97) $$\lim_{n\to \infty }\frac{1}{n}U_{n}^{2}=\lim_{n\to \infty }\frac{∥R+\sqrt{s}{Z∥}^{2}}{n}=s.$$

Now, combining the capacity expression in (58) and (A96), we have that

(A98) $$\begin{array}{c} \lim_{n\to \infty }C_{s}\left(\sigma_{1}^{2},\sigma_{2}^{2},R,n\right)=\frac{1}{2}\int_{\sigma_{1}^{2}}^{\sigma_{2}^{2}}\frac{R^{2}}{s^{2}}ds=R^{2}\left(\frac{1}{2\sigma_{1}^{2}}-\frac{1}{2\sigma_{2}^{2}}\right). \end{array}$$

## Appendix D. Proof of Theorem 8

Let $R_{n}=c\sqrt{n}$

(A99) $$\begin{array}{cc} \lim_{n\to \infty }\frac{C_{s}\left(\sigma_{1}^{2},\sigma_{2}^{2},R_{n},n\right)}{n} & =\frac{c^{2}}{2}\int_{\sigma_{1}^{2}}^{\sigma_{2}^{2}}\frac{1-\lim_{n\to \infty }E\left[h_{\frac{n}{2}}^{2}\left(\frac{∥R_{n}+\sqrt{s}Z∥R_{n}}{s}\right)\right]}{s^{2}}ds \end{array}$$

(A100) $$\begin{array}{cc} \; & =\frac{c^{2}}{2}\int_{\sigma_{1}^{2}}^{\sigma_{2}^{2}}\frac{1-\frac{c^{2}\left(c^{2}+s\right)}{{\left(\frac{s}{2}+\sqrt{\frac{s^{2}}{4}+c^{2}\left(c^{2}+s\right)}\right)}^{2}}}{s^{2}}ds \end{array}$$

(A101) $$\begin{array}{cc} \; & =\frac{1}{2}\log\left(\frac{\sigma_{2}^{2}\left(c^{2}+\sigma_{1}^{2}\right)}{\sigma_{1}^{2}\left(c^{2}+\sigma_{2}^{2}\right)}\right), \end{array}$$

where (A100) follows from the limit established in (98). This concludes the proof.

## Appendix E. Partial Derivatives for the Gradient Ascent Algorithm

The partial derivatives of the secrecy information, with respect to any mass point $\rho_{l}\in \mathrm{supp}\left(P_{∥X∥}\right)$, are defined as

(A102) $$\begin{array}{cc} \frac{\partial }{\partial \rho_{l}}I_{s}(∥X∥;P_{∥X∥}) & =\sum_{k=1}^{K}p_{i}\cdot \frac{\partial }{\partial \rho_{l}}\tilde{\Xi }\left(\rho_{k}\;;{\hat{P}}_{∥X∥}\right),\;l=1,\cdots ,K. \end{array}$$

By (A9), we have that $\tilde{\Xi }\left(∥x∥\;;{\hat{P}}_{∥X∥}\right)=i_{1}(∥x∥;P_{X})-i_{2}(∥x∥;P_{X})$, where $i_{j}(∥x∥;P_{X})$, for j=1,2, is defined in (A10). Therefore, to compute (A102), we define the following derivatives

(A103) $$\begin{array}{cc} \frac{\partial }{\partial \rho_{l}}i_{j}\left(\rho_{k};P_{X}\right) & =\int_{0}^{\infty }\frac{\partial }{\partial \rho_{l}}\left(f_{\chi_{n}^{2}\left(\rho_{k}^{2}/\sigma_{j}^{2}\right)}\left(y\right)\log\frac{y^{\frac{n}{2}-1}}{\sum_{m=1}^{K}p_{m}f_{\chi_{n}^{2}\left(\rho_{m}^{2}/\sigma_{j}^{2}\right)}\left(y\right)}\right)dy, \end{array}$$

where $f_{\chi_{n}^{2}\left(\rho_{k}^{2}/\sigma_{j}^{2}\right)}\left(y\right)$ is the noncentral chi-square pdf with noncentrality parameter $\rho_{k}^{2}/\sigma_{j}^{2}$ and *n* degrees of freedom. Notice that the derivative of $f_{\chi_{n}^{2}\left(\rho_{k}^{2}/\sigma_{j}^{2}\right)}\left(y\right)$ with respect to $\rho_{l}$ is different from zero only when k=l and is given by

(A104) $$\begin{array}{cc} \frac{\partial }{\partial \rho_{l}}f_{\chi_{n}^{2}\left(\rho_{l}^{2}/\sigma_{j}^{2}\right)}\left(y\right) & =\frac{\rho_{l}}{\sigma_{j}^{2}}\left(f_{\chi_{n+2}^{2}\left(\rho_{l}^{2}/\sigma_{j}^{2}\right)}\left(y\right)-f_{\chi_{n}^{2}\left(\rho_{l}^{2}/\sigma_{j}^{2}\right)}\left(y\right)\right). \end{array}$$

Moreover, given the probability $p_{l}$ associated with $\rho_{l}$, we have that

(A105) $$\begin{array}{cc} \frac{\partial }{\partial \rho_{l}}\log\frac{y^{\frac{n}{2}-1}}{\sum_{k=1}^{K}p_{k}f_{\chi_{n}^{2}\left(\rho_{k}^{2}/\sigma_{j}^{2}\right)}\left(y\right)} & =-p_{l}\frac{\frac{\partial }{\partial \rho_{l}}f_{\chi_{n}^{2}\left(\rho_{l}^{2}/\sigma_{j}^{2}\right)}\left(y\right)}{\sum_{k=1}^{K}p_{k}f_{\chi_{n}^{2}\left(\rho_{k}^{2}/\sigma_{j}^{2}\right)}\left(y\right)}. \end{array}$$

Finally, by combining everything together, we find

(A106) $$\begin{array}{cc} \; & \frac{\partial }{\partial \rho_{l}}I_{s}(∥X∥;P_{∥X∥})=\\ \; & \;p_{l}\int_{0}^{\infty }\frac{\rho_{l}}{\sigma_{1}^{2}}\left(f_{\chi_{n+2}^{2}\left(\rho_{l}^{2}/\sigma_{1}^{2}\right)}\left(y\right)-f_{\chi_{n}^{2}\left(\rho_{l}^{2}/\sigma_{1}^{2}\right)}\left(y\right)\right)\left[\log\frac{y^{\frac{n}{2}-1}}{\sum_{k=1}^{K}p_{k}f_{\chi_{n}^{2}\left(\rho_{k}^{2}/\sigma_{1}^{2}\right)}\left(y\right)}-1\right]dy\\ \; & \;-p_{l}\int_{0}^{\infty }\frac{\rho_{l}}{\sigma_{2}^{2}}\left(f_{\chi_{n+2}^{2}\left(\rho_{l}^{2}/\sigma_{2}^{2}\right)}\left(y\right)-f_{\chi_{n}^{2}\left(\rho_{l}^{2}/\sigma_{2}^{2}\right)}\left(y\right)\right)\left[\log\frac{y^{\frac{n}{2}-1}}{\sum_{k=1}^{K}p_{k}f_{\chi_{n}^{2}\left(\rho_{k}^{2}/\sigma_{2}^{2}\right)}\left(y\right)}-1\right]dy. \end{array}$$
